# Dual-mode optical temperature sensing using Dy^3+^/Sm^3+^ co-activated Ba_2_ZnSi_2_O_7_ phosphor with tuneable sensitivity

**DOI:** 10.1039/d5ra09381c

**Published:** 2026-02-16

**Authors:** Tejas, A. Princy, S. Masilla Moses Kennedy, Sudha D. Kamath

**Affiliations:** a Manipal Institute of Technology, Manipal Academy of Higher Education Manipal Karnataka India sudha.kamath@manipal.edu; b Sri Siva Subramaniya Nadar College of Engineering Tamil Nadu India

## Abstract

This study reports the synthesis and characterization of a novel Ba_2_ZnSi_2_O_7_:Dy^3+^, Sm^3+^ phosphor designed for optical temperature sensing applications. The material was successfully prepared using a high-temperature solid-state reaction method. X-ray diffraction (XRD) confirmed a monoclinic crystal structure with high phase purity. Photoluminescence (PL) spectroscopy identified 1 mol% Sm^3+^ as the optimal doping concentration for efficient luminescence, and significant energy transfer from Dy^3+^ to Sm^3+^ was observed and quantified. Diffuse reflectance spectroscopy (DRS) indicated a widened bandgap due to a shift in the conduction band upon co-doping. Scanning electron microscopy (SEM) revealed an agglomerated morphology, while FTIR analysis confirmed the structural integrity of the host lattice after doping. Under 403 nm excitation, the activation energy for thermal quenching of Sm^3+^ emission was determined to be 0.19 eV. The fluorescence intensity ratio (*I*_Dy_/*I*_Sm_) displayed strong temperature dependence between 303 K and 483 K, achieving a maximum relative sensitivity of 7.19% K^−1^ at 403 K. Additionally, Sm^3+^ lifetime measurements showed a high temperature-dependent sensitivity, with a maximum of 1.10% K^−1^ at 303 K. These results highlight the material's excellent potential for high-performance optical temperature sensing.

## Abstract 2

This study reports the synthesis and characterization of a novel Ba_2_ZnSi_2_O_7_:Dy^3+^, Sm^3+^ phosphor designed for optical temperature sensing applications.

## Graphical abstract

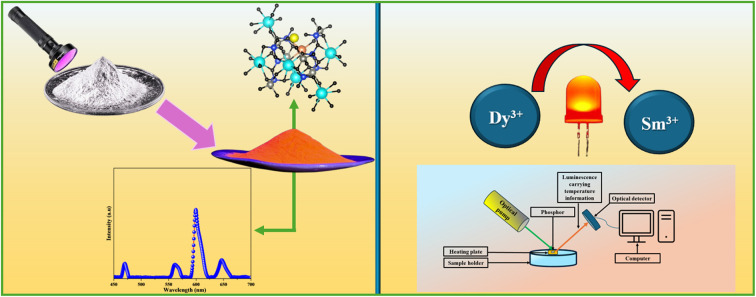

## 1. Introduction

Temperature, as a fundamental thermodynamic factor, plays a crucial role in the functioning and durability of both natural and engineered systems, across all scales. Even small fluctuations in temperature can have a significant impact on various engineering and biological activities.^1,2^ Consequently, precise and reliable temperature measurements are vital in numerous scientific and technological areas. It is therefore unsurprising that temperature sensors account for approximately 80% of the global sensor market, which, according to Grand View Research, is projected to reach a value of 8.0 USD billion by 2028.^3^

Currently, non-contact thermal sensing techniques that utilize changes in the optical properties of materials are considered among the most promising methods for temperature measurement. Various optical approaches, including infrared thermography, pyrometry, and luminescent thermometry, have been explored for developing advanced thermal sensors.^4,5^ While thermography is widely used, it is an expensive and complex technique with notable limitations.^6,7^ For example, it only measures surface temperature, even for transparent objects in the visible spectrum, and its accuracy depends heavily on the emissivity of the object. Similarly, pyrometers are limited by a relatively low accuracy in temperature readings, typically around ± 5 °C.^8^ In contrast, luminescence thermometry overcomes these challenges, offering exceptional precision in both temperature measurements and mapping of temperature distribution. This method provides continuous, real-time thermal readings with high spatial and temperature resolution, and it is versatile, resistant to electromagnetic interference, and capable of functioning in harsh environments.^9^ Luminescence thermometry works by monitoring changes in luminescent properties such as emission intensity, intensity ratios, decay time, shifts in the peak luminescence wavelength, or variations in the bandwidth.^10–13^

Rare-earth (RE) luminescent materials have garnered significant interest due to their diverse applications in fields such as artificial lighting, X-ray medical imaging, lamps, display technologies, and high-power solid-state lasers.^14^ Among these, RE doped silicates are highly regarded as host materials due to their excellent chemical stability, adequate thermal conductivity, and moderate light emission. Thermographic phosphors (TGPs) are specialized phosphors designed for use in phosphor thermometry. Research in phosphor thermometry typically focuses on two primary methods: lifetime decay method^15,16^ and the intensity ratio method.^17–19^ The intensity ratio method has gained more widespread use, as it helps eliminate noise caused by fluctuations in the excitation light source, temperature-induced changes in excitation bands, and variations in dopant concentrations.^20^ RE ions with 4f^n^ electronic configurations having a weak electron–lattice interaction with different quenching channels may induce significant temperature-sensitive FIR to get a higher temperature sensitivity.^14^ Dysprosium (Dy^3+^) and Samarium (Sm^3+^) were doped into host to get higher sensitivity. The temperature-dependent fluorescence lifetime of Dy^3+^ employed as lifetime-mode optical thermometry should also be investigated to explore its latent applications in bioscience. This means if we co-dope Dy^3+^ ions with one kind of RE ions into host, we may obtain a kind of phosphor with dual-mode temperature sensing function. Samarium has a complex energy level structure, with ground state ^6^H_*J*_ and ^6^F_*J*_ multiplets, along with an excited ^4^G_5/2_ level, enabling fluorescence in the visible and near-infrared regions. The emission of Sm^3+^ ions from intra-4f shell transitions is highly efficient, making them crucial in luminescent processes.^21,22^ Among these, there are some of the reported works Ba_2_ZnSi_2_O_7_:Eu^3+^, B^3+^,^23^ Ba_2_ZnSi_2_O_7_:Eu^2+^, Dy^3+^,^24^ Ba_2_ZnSi_2_O_7_:Ce^3+^, Eu^3+^, Eu^2+^,^25^ and Ba_2_ZnSi_2_O_7_:Ce^3+^, Tb^3+^.^26^ Based on the existing literature, previous studies on Ba_2_ZnSi_2_O_7_ based phosphors have primarily focused on LED applications with various dopant combinations. However, to the best of our knowledge, no work has been reported on the use of Ba_2_ZnSi_2_O_7_:Dy^3+^, Sm^3+^ for optical thermometry. This study presents the first investigation of this dual-doped system for temperature sensing, highlighting its novel application and promising performance in optical thermometry.

In this work, Ba_2_ZnSi_2_O_7_:Dy^3+^, Sm^3+^ phosphors were synthesized using the solid-state reaction method. Various characterization techniques were employed to explore and analyze their optical, thermal, structural, and morphological properties. We conducted a detailed examination of the thermally stimulated delayed photoluminescence and room-temperature photoluminescence (PL) spectra. Additionally, we investigated the nonradiative cross-relaxation mechanism, energy transfer processes, thermal stability, and temperature-dependent photoluminescence characteristics, particularly focusing on their potential applications in non-contact optical thermometry.

## 2. Experimental details

### 2.1. Methodology

The phosphor was synthesized through a solid-state reaction method using high-purity reagents BaCO_3_ (barium carbonate, 99%), ZnO (zinc oxide, 99%), SiO_2_ (silicon dioxide, 99%), Dy_2_O_3_ (dysprosium oxide, 99.99%), and Sm_2_O_3_ (samarium oxide, 99.99%). The desired stoichiometric amounts of these chemicals were carefully weighed and mixed in a mortar. The stoichiometry was taken from balanced chemical equation given below,1
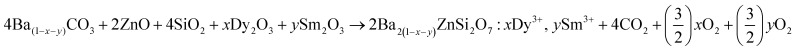


The mixture was ground for 1 hour to ensure thorough homogeneity of the components. After grinding, the powder was transferred to an alumina crucible and subjected to a high-temperature treatment to promote the solid-state reaction. The heating process was carried out in a muffle furnace, with a heating rate of 4 °C min^−1^ to a target temperature of 1200 °C. The sample was held at 1200 °C for 6 hours to allow complete reaction and formation of the phosphor phase. After the heat treatment, the synthesized material was allowed to cool to room temperature inside the furnace. The resulting phosphor was then collected and characterized for further analysis. The detailed study of the Dy^3+^ ions in Ba_2_ZnSi_2_O_7_ host was explored. The concentration of dysprosium ions was optimized in our previous reported work.^27^ The optimized concentration of Dy^3+^ ion was 1.5 mol% which is fixed in this study and varied the concentration of Sm^3+^ ions to study their properties.^27^

### 2.2. Characterization techniques

The crystal structure of the synthesized sample was determined using X-ray diffraction (XRD) analysis. The measurements were performed on a Rigaku MiniFlex 600 X-ray diffractometer (5th generation), employing nickel-filtered Cu K_α_ radiation (*λ* = 1.540 Å) over a 2*θ* range of 20° to 80°. The lattice parameters were calculated using the FullProf suite of programs. Scanning electron microscopy (SEM) imaging and energy dispersive spectroscopy (EDS) were conducted using a ZEISS EVO MA18 SEM equipped with an Oxford X-act detector for morphological and compositional analysis. Diffuse reflectance spectra (DRS) were recorded using a PerkinElmer Lambda 950 UV-vis spectrophotometer. Fourier transform infrared (FTIR) spectra were collected on a Shimadzu FTIR instrument. Photoluminescence excitation (PLE) and photoluminescence (PL) spectra were obtained using a Jasco FP-8500 spectrofluorometer with a Xenon flash lamp as the excitation source. Thermal analysis was performed with a PerkinElmer TGA 4000 thermogravimetric analyzer. For temperature-dependent photoluminescence lifetime and emission spectra, an Agilent Cary Eclipse Fluorescence Spectrophotometer with an integrated heater was utilized.

## 3. Results and discussion

### 3.1. X-ray diffraction crystal structure studies

**Fig. 1:**
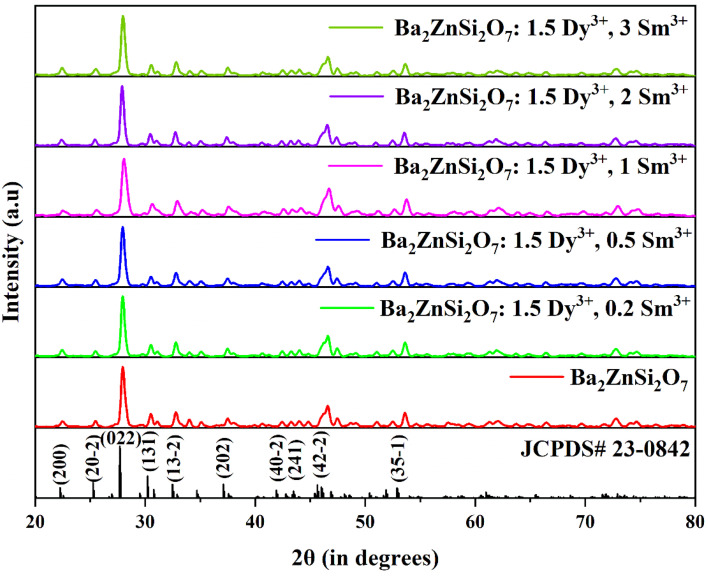
XRD patterns of Ba_2_ZnSi_2_O_7_:1.5 mol% Dy^3+^, *y*Sm^3+^ (*y* = 0.2, 0.5, 1, 2, 3 mol%) with standard card of Ba_2_ZnSi_2_O_7_ as a reference.

The X-ray diffraction patterns for the Ba_2_ZnSi_2_O_7_:1.5 mol% Dy^3+^ phosphors co-doped with varying concentrations of Sm^3+^ (*y* = 0.2, 0.5, 1, 2, 3 mol%) are shown in Fig. 1. The results clearly reveal that all the diffraction peaks match those of the standard Ba_2_ZnSi_2_O_7_ JCPDS card 23-0842, confirming that the Ba_2_ZnSi_2_O_7_ has been successfully synthesized.^28^ Furthermore, the doping of Dy^3+^ and Sm^3+^ does not alter the crystal phase of Ba_2_ZnSi_2_O_7_. As seen in Fig. 1, there are no additional peaks, indicating that the incorporation of Dy^3+^ and Sm^3+^ does not cause any major structural changes to the Ba_2_ZnSi_2_O_7_ crystal but we can observe small shift in the XRD pattern that might be due to the incorporation of dopants into host sites. Although XRD is an effective method for identifying phases, it should be noted that it might not be able to identify small impurity phases below about 2–3 weight percent. The wide-angle scan's lack of unknown peaks and strong match to the reference phase, however, indicate that any such impurities if any are below the detection threshold and unlikely to have a substantial impact on the optical characteristics. The phase purity of the produced samples is further supported by the regulated synthesis procedure, ideal stoichiometry, and steady photoluminescence activity. To understand the unchanged XRD patterns after doping, we calculate the allowed radius percentage difference (*D*_R_). This value indicates the maximum permissible difference in ionic radii for a successful substitution of the dopant ion. The *D*_R_ threshold is typically set at 30%, meaning that if the calculated *D*_R_ value is below this limit, the dopant ion is likely to replace the host ion. The *D*_R_ percentage is determined using a specific formula,^29^2
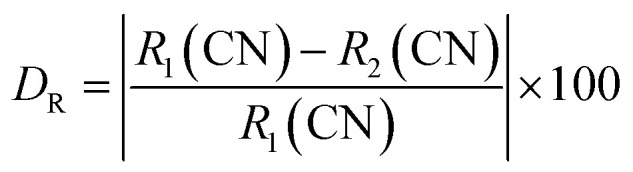


The ionic radius of the dopant is represented as *R*_2_, while the ionic radius of the host cation is *R*_1_, and CN refers to the coordination number of the ions. By using the atomic radii of both the host and dopant ions, we have calculated the *D*_R_ values for various combinations. The results of these calculations are presented in Table S1.

Table S1 clearly shows that *D*_R_ values for Ba^2+^ and Dy^3+^ ranges from 0.74% to 51.93% and Ba^2+^ and Sm^3+^ ranging from 5.67% to 49.82%. Based on the results, it can be concluded that replacing Ba^2+^ (atomic radius of 1.35 Å) with Dy^3+^ (atomic radius 1.02 Å) and Sm^3+^ (atomic radius 1.07 Å) is feasible, as the *D*_R_ values for these substitutions are significantly low.^30^

**Fig. 2:**
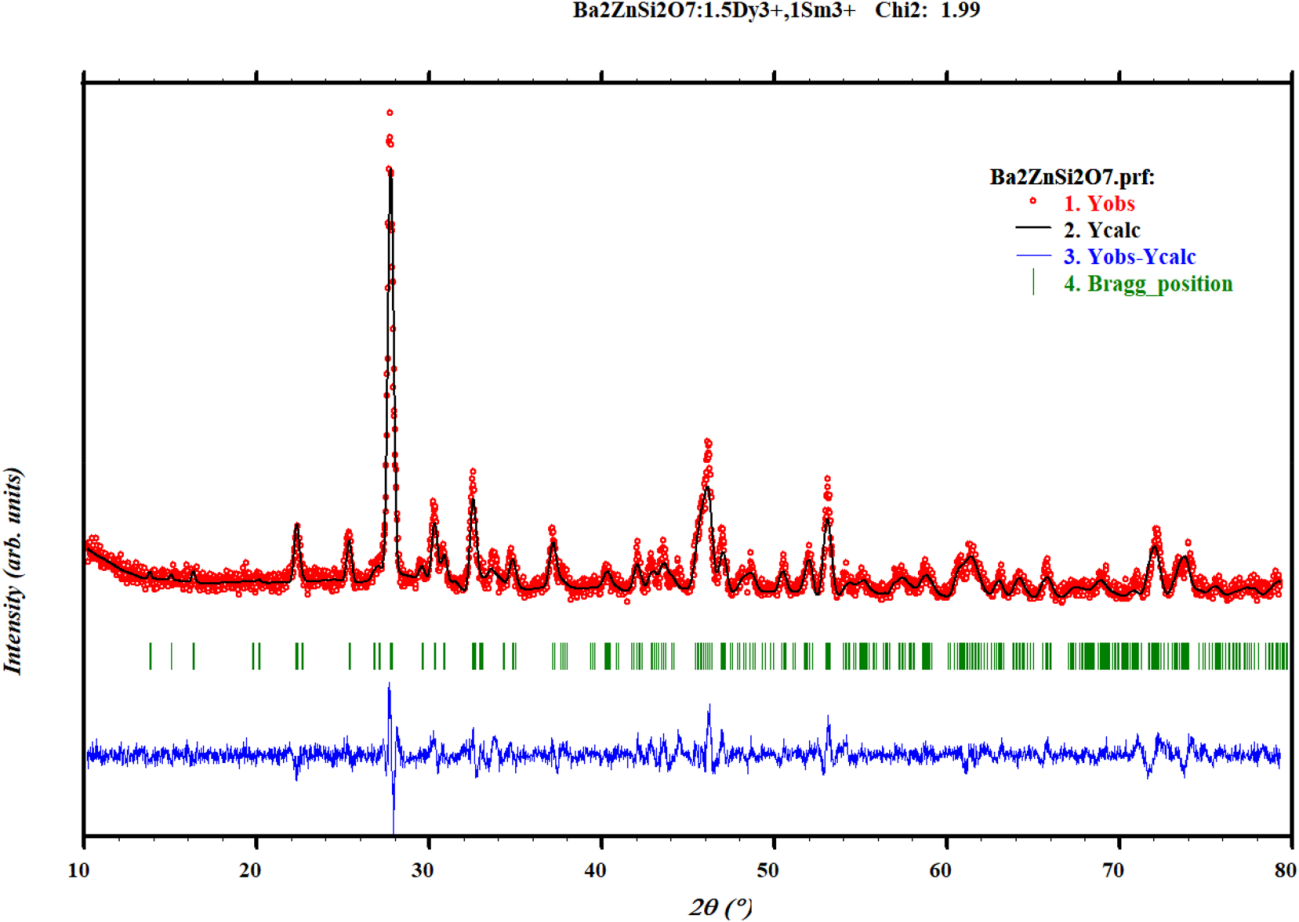
Rietveld refinement for the Ba_2−*x*−*y*_ZnSi_2_O_7_:1.5 mol% Dy^3+^, *y*Sm^3+^ (*y* = 1 mol%) phosphor.

We performed Rietveld refinement on the Ba_2−*x*−*y*_ZnSi_2_O_7_:*x*Dy^3+^, *y*Sm^3+^ (*x* = 1.5, *y* = 1 mol%) phosphor to analyze its crystal structure. The refinement yielded a convergence with *R*_wp_ = 26.2%, *R*_p_ = 26%, and *χ*^2^ = 1.66, confirming that Dy^3+^ and Sm^3+^ ions have successfully been incorporated into the host lattice. Table S2 shows the refined structural parameters. The refined structural parameters are as follows: *α* = *γ* = 90°, *β* = 110.92°, and lattice dimensions *a* = 8.5161 Å, *b* = 10.8727 Å, *c* = 8.5612 Å and *V* = 740.4184 Å^3^. The Rietveld refinement of the optimized phosphor is shown in Fig. 2. The refined parameters are listed in Table 1. The crystal structure of Ba_2_ZnSi_2_O_7_:1.5 Dy^3+^, 1 Sm^3+^ belongs to the *C*2/*c* space group and has a monoclinic configuration.^31^ The crystal structure can be visualized using VESTA software.

**Table 1:** Refined parameters of Ba_2_ZnSi_2_O_7_:1.5 Dy^3+^, 1 Sm^3+^ phosphors

Sample	Ba_2−0.015−0.010_ZnSi_2_O_7_:1.5 Dy^3+^, 1 Sm^3+^
Symmetry	Monoclinic
Space group	*C*2/*c*
Centrosymmetric	Centric
*a* (Å)	8.5199
*b* (Å)	10.8548
*c* (Å)	8.5369
*α* (°)	90.000
*β* (°)	110.9055
*γ* (°)	90.000
*Z*	8
*V* (Å^3^)	737.5372
Density (g cm^−3^)	4.577
*R*_P_	11.8
*R*_WP_	14.8
*χ* ^2^	1.99

**Fig. 3:**
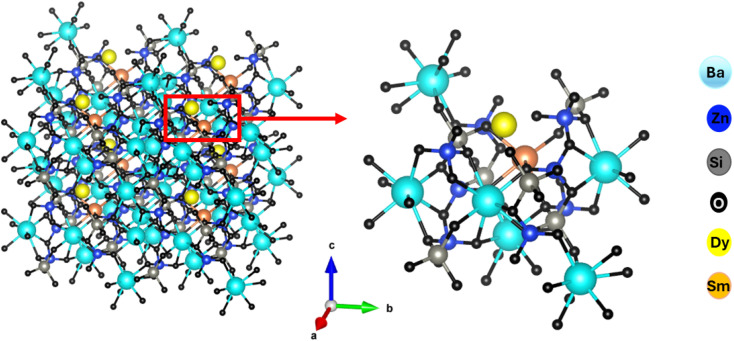
3D visualization of Ba_2_ZnSi_2_O_7_:Dy^3+^, Sm^3+^ phosphors.

Fig. 3 shows the crystal structure of Ba_2_ZnSi_2_O_7_:Dy^3+^, Sm^3+^ phosphor. The crystallite size and lattice strain are determined using the Debye–Scherrer method, a straightforward technique for analysing peak broadening and calculating crystallite size. To find the average crystallite size, the Full Width at Half Maximum (FWHM) value of the main peaks is used in the calculation, following eqn (3),^32^3
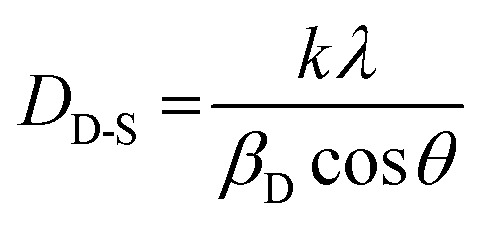


The crystallite size (*D*_D-S_) is calculated using a formula where *k* is the shape factor, valued at 0.9, *λ* is the wavelength of Cu-K_α_ radiation, set at 1.54 Å, *β*_D_ represents the Full Width at Half Maximum (FWHM), and *θ* is the diffraction angle. The estimated crystallite sizes are provided in Table S3. To analyze the isotropic crystal structure, the size–strain approach is used.^33^ This method also helps estimate the size and strain of the particles. The Size–Strain Plot (SSP) analysis shown in Fig. S1, the lattice strain is explained by a Gaussian function, while the crystallite size is clarified by a Lorentzian function.^34^4
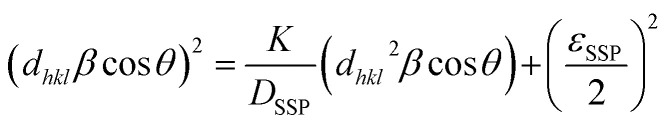


By plotting (*d*_*hkl*_β cos *θ*)^2^ against (*d*_*hkl*_^2^β cos *θ*) as shown in Fig. S1, the crystallite size and apparent lattice strain are determined from the slope and intercept of the linearly extrapolated data, respectively. These values are then listed in Table S3. We can observe that through both methods the average crystallite size was found to be 0.0211 µm ≈ 21 nm. Average is also found to be 0.0165.

### 3.2. Photoluminescence studies

**Fig. 4:**
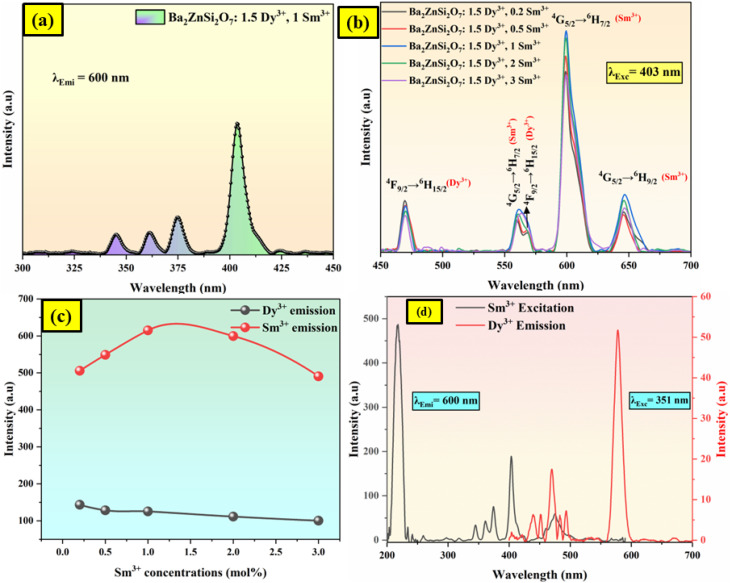
(a) PL excitation spectra (b) PL emission spectra of Ba_2_ZnSi_2_O_7_:1.5 mol% Dy^3+^, *y*Sm^3+^ (*y* = 0.2, 0.5, 1, 2, 3 mol%) phosphors (c) intensity variation *versus* dopant concentrations (d) spectral overlap between the excitation spectrum of Ba_2_ZnSi_2_O_7_:1 mol% Sm^3+^ and the emission spectrum of Ba_2_ZnSi_2_O_7_:1.5 mol% Dy^3+^.

We have doped Dy^3+^ ions in Ba_2_ZnSi_2_O_7_ matrix with different concentrations ranging from 1 to 2 mol% and 1.5 mol% was found to be optimum concentration which is reported in our previous work.^27^ To the optimized concentration of Dy^3+^, we co-doped different concentration of Sm^3+^ (*y* = 0.2, 0.5, 1, 2, and 3 mol%) in Ba_2_ZnSi_2_O_7_ matrix. The room temperature PL excitation spectra is shown in Fig. 4(a). The excitation spectra comprise of four different peaks 345, 361, 375 and 403 nm corresponding to ^6^H_5/2_ → ^4^H_9/2,_^6^H_5/2_ → ^4^D_3/2_, ^6^H_15/2_ → ^6^P_7/2_ and ^6^H_15/2_ → ^4^P_3/2_.^35^ Among all the identified peaks, the excitation peak with the strongest intensity was observed at 403 nm. This peak corresponds to the transition of Sm^3+^ ions from the ^6^H_5/2_ state to the ^4^P_3/2_ state. Therefore, to obtain the emission spectra for each synthesized phosphor, we chose 403 nm as the excitation wavelength. Fig. 4(b) shows the PL emission of Ba_2_ZnSi_2_O_7_:1.5 mol% Dy^3+^, *y*Sm^3+^ (*y* = 0.2, 0.5, 1, 2, 3 mol%) phosphors when excited at 403 nm. The emission spectra show 470, 560, 571, 600, and 646 nm corresponding to the transitions ^4^F_9/2_ → ^6^H_13/2_, ^4^G_5/2_ → ^6^H_5/2_, ^4^F_9/2_ → ^6^H_15/2_, ^4^G_5/2_ → ^6^H_7/2_ and ^4^G_5/2_ → ^6^H_9/2_ respectively.^35,36^ Out of these, 470 and 571 nm are due to Dy^3+^ ions. The most intense transition is the ^4^G_5/2_ → ^6^H_7/2_ at 600 nm and follows the Δ*J* = ±1 selection rule. It is well known that electric dipole transitions obey the selection rules Δ*J* ≤ 6, if *J* or *J*′ = 0 and Δ*J* = 2, 4, or 6 otherwise. Magnetic dipole transitions obey the selection rule Δ*J* = 0 or ±1. The ^4^G_5/2_ → ^6^H_5/2_ transition is an allowed magnetic dipole (MD) transition, the ^4^G_5/2_ → ^6^H_7/2_ transition is a mixed transition and the ^4^G_5/2_ → ^6^H_9/2_ transition is a pure electric dipole (ED) transition.^37^ It implies that the Sm^3+^ ions were more symmetric in the host matrix because the observed MD transition of Sm^3+^ ion (^4^G_5/2_ → ^6^H_7/2_) is stronger than the observed ED transition of Sm^3+^ ion and Dy^3+^ ions in the present study.^38^ The higher intensity ratio values indicate higher distortion from the inversion symmetry. The intensity variation can be seen in Fig. 4(c). We establish that the transition in the emission spectra from ^4^G_5/2_ → ^6^H_9/2_ with a centre at 600 nm is the most intense and therefore the primary contributor to the reddish-orange emission emitted from the phosphor.

#### 3.2.1. Energy transfer analysis

To optimize the emission color of phosphor, the co-doping of Dy^3+^ and Sm^3+^ were explored based on the interaction of the two-ion doping, of which the energy transfer was the main mechanism. The spectral overlap of the sensitizer and activator is one of the main metrics of energy transfer. The spectral overlap between the excitation spectrum of Ba_2_ZnSi_2_O_7_:1 mol% Sm^3+^ and the emission spectrum of Ba_2_ZnSi_2_O_7_:1.5 mol% Dy^3+^ was plotted as shown in Fig. 4(d). The results clearly show significant spectral overlap between the Dy^3+^ emission band of transition ^4^F_9/2_ → ^6^H_15/2_ and the Sm^3+^ excitation band of transition ^5^H_5/2_ → ^4^M_15/2_ in the 400–500 nm range. The Dexter energy transfer theory states that Dy^3+^ ions can easily transfer energy to Sm^3+^ ions as a non-radiative process in the co-doped incandescent samples, improving the resultant fluorescence emission of Sm^3+^ ions.^39^ Referring to Fig. 4(b), it can be observed that with the increase of Sm^3+^ co-dopant concentration from 0.2 to 1 mol%, the intensity of the characteristic emission peaks of Dy^3+^ at 470 nm, and 571 nm decreases monotonically while that of Sm^3+^ at 560, 600 nm and 646 nm increases gradually. Later on, doping Sm^3+^ more than 1 mol% the Sm^3+^ emission decreases while the Dy^3+^ emission was getting decreased due to the concentration quenching. The optimum concentration of Sm^3+^ ions was found to be 1 mol%.

The energy transfer pathway of Dy^3+^ → Sm^3+^ is further supported by the prior discussion. The concentration quenching effect causes the characteristic emission intensity of Sm^3+^ to gradually diminish when the Sm^3+^ doping concentration rises over 1 mol%. For concentration quenching there are two major factors which are quenching centers and cross relaxation. In concentration quenching the distance between active ions reduces with increasing doping concentration. So, energy transfer between neighbouring activated ions takes place when this distance falls below the critical distance (*R*_c_). Transferring the energy to a quenching center will result in fluorescence quenching since it will be released by a non-radiative transition that does not contribute to luminescence. Secondly, cross-relaxation can happen between the same ions or between distinct ions when the energy level separation between them is tiny. Energy transfer through non-radiative transitions may result from this, which might extinguish fluorescence and cause energy loss. Furthermore, the crystals impurity defects and structural flaws such as surface or point defects may function as non-radiative centers. The energy lost as heat when electrons are trapped by these imperfections also plays a role in the quenching of fluorescence. We used the eqn (5) to determine the critical distance *R*_c_ in order to fully comprehend the energy transfer phenomena in the co-doping system,^40^5
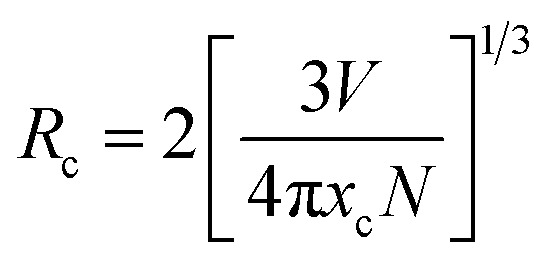
Here, *N* is the number of cations in the unit cell, *x*_c_ is the critical concentration, and *V* is the unit cell volume. The following values were determined for the Ba_2_ZnSi_2_O_7_ system: *V* = 740.4184 Å^3^, *N* = 4, *x*_c_ ≈ 0.01, and *R*_c_ = 32.82. The energy transfer mechanism may be divided into two categories according to the *R*_c_ value as multipole interactions which are the primary determinant when *R*_c_ is larger than 5 Å, whereas exchange interactions predominate when *R*_c_ is less than 5 Å. Thus, multipole interaction plays a major role in energy transfer in the Ba_2_ZnSi_2_O_7_:Dy^3+^/Sm^3+^ system. Further, Dexter's theory and Reisfeld's approximation may be used to gain more understanding of the multipole interaction process.^41,42^6
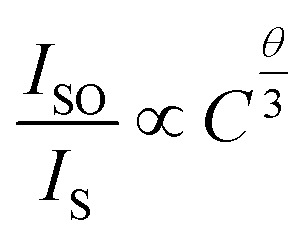


The fluorescence intensity of the single-doped Dy^3+^ is represented by *I*_SO_, the fluorescence intensity of the co-doped Dy^3+^ and Sm^3+^ by *I*_S_, the total doping concentration of Dy^3+^ and Sm^3+^ by *C*, and the dipole–dipole, dipole-quadrupole, and quadrupole–quadrupole energy transfer modes, respectively, by *θ*, which can be 6, 8, or 10. Dipole–dipole interactions are probably the predominant mechanism in the energy transfer process from Dy^3+^ to Sm^3+^, as seen in Fig. S2(a–c), where the best match occurs when *θ* = 6, the fitted value matches to 0.95.

**Fig. 5:**
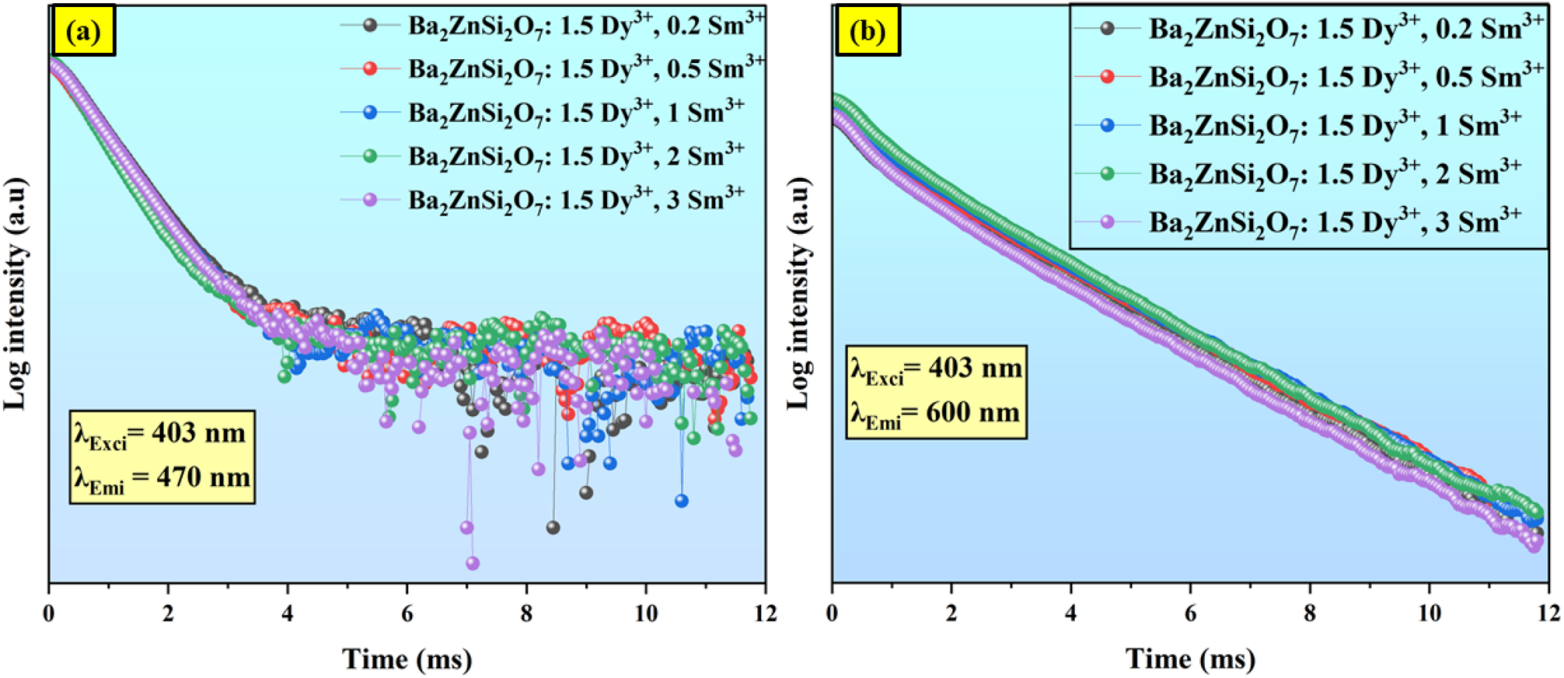
Fluorescence decay curve of Ba_2_ZnSi_2_O_7_:1.5 mol% Dy^3+^, *y*Sm^3+^ (*y* = 0.2, 0.5, 1, 2, 3 mol%) with (a) *λ*_ex_ = 403 nm and *λ*_em_ = 470 nm (b) *λ*_ex_ = 403 nm and *λ*_em_ = 600 nm.

The fluorescence decay behaviour of Dy^3+^ emission at 470 nm (^4^F_9/2_ → ^6^H_13/2_) is analyzed using the fluorescence decay curve of Ba_2_ZnSi_2_O_7_:1.5 mol% Dy^3+^, *y*Sm^3+^ (*y* = 0.2, 0.5, 1, 2, 3 mol%) which is excited at *λ*_ex_ = 403 nm and emitted at *λ*_em_ = 470 nm. In Fig. 5(a), this behaviour is seen. A single exponential function fits all of the decay curves in Fig. 5(a) well. Similarly, we examined the fluorescence lifetime curves of Ba_2−*x*−*y*_ZnSi_2_O_7_:1.5 mol% Dy^3+^, *y*Sm^3+^ (where *y* = 0.2, 0.5, 1.0, 2.0, 3.0 mol%), phosphors at 600 nm under 403 nm excitation as shown in Fig. 5(b). A single exponential decay pattern was seen in the fluorescence lifetime curves,^43^7
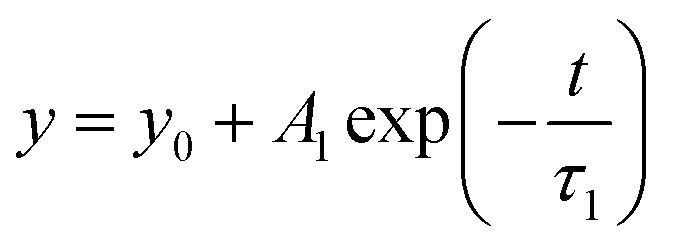
8
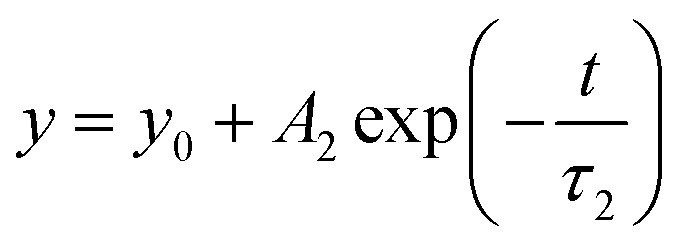
where *y* is the luminescence intensity at time *t*, *A*_1_ and *A*_2_ are constants, *τ*_1_ is the lifetime of 470 nm emission when excited at 403 nm and *τ*_2_ is the lifetime of 600 nm emission when excited at 403 nm. The lifetime values of Sm^3+^ ions and Dy^3+^ ions in Ba_2_ZnSi_2_O_7_ matrix are listed in Table 2.

**Table 2:** Lifetimes and energy transfer efficiencies of Ba_2−*x*−*y*_ZnSi_2_O_7_:*x*Dy^3+^, *y*Sm^3+^ phosphors

Dy^3+^ concentration (mol%)	Sm^3+^ concentration (mol%)	*τ*_1_ (ms)	*τ*_2_ (ms)	Efficiency (%)
1.5	0	0.4878 ± 0.0053	—	—
1.5	0.2	0.4656 ± 0.0043	1.7038 ± 0.019	72.66 ± 0.40
1.5	0.5	0.4287 ± 0.0044	1.7664 ± 0.016	75.72 ± 0.34
1.5	1	0.4424 ± 0.0045	1.7892 ± 0.015	75.27 ± 0.32
1.5	2	0.4026 ± 0.0046	1.7288 ± 0.014	76.70 ± 0.33
1.5	3	0.3870 ± 0.0057	1.7046 ± 0.013	77.29 ± 0.38

The fluorescence lifetime of Dy^3+^ gradually decreases as the concentration of Sm^3+^ rises while the concentration of Dy^3+^ stays constant. The existence of energy transfer between Dy^3+^ and Sm^3+^ ions is further supported by this discovery. The following is an expression for the energy transfer efficiency between the activator ion (Sm^3+^) and the sensitizer (Dy^3+^),^44^9
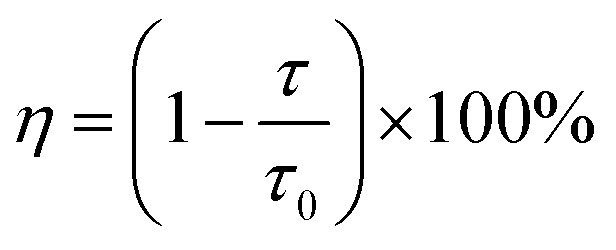


**Fig. 6:**
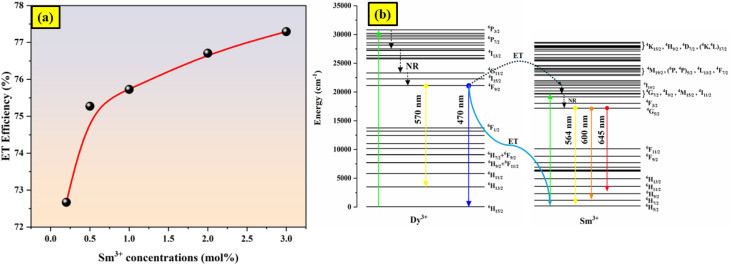
(a) Variation of energy transfer efficiency (b) energy level diagrams of Dy^3+^–Sm^3+^.

*τ* and *τ*_0_ stand for the Dy^3+^ emission intensities with and without Sm^3+^ ions, respectively. For the excitation light at 403 nm, the *τ* and *τ*_0_ values are summed to determine the energy transfer efficiency (*η*) which is listed in Table 2. The energy transfer efficiency from Dy^3+^ to Sm^3+^ improves from 72% to 77% as the concentration of Sm^3+^ climbs from 0.2 to 3 mol%. These findings demonstrate that during the Dy^3+^ → Sm^3+^ transition, there is efficient energy transfer between Dy^3+^ and Sm^3+^ ions. Fig. 6(a) shows the variation of energy transfer efficiency with respect to varied concentrations.

As seen in Fig. 6(b), the energy transition process in the Ba_2−*x*−*y*_ZnSi_2_O_7_:*x*Dy^3+^, *y*Sm^3+^ phosphor system describes the mechanism of energy transfer between Dy^3+^ and Sm^3+^ ions. Dy^3+^ ions absorb energy when the system is activated by UV light at 403 nm, which moves its electrons from the ground state ^6^H_15/2_ to an excited state ^4^P_3/2_. The lowest excited state (^4^F_9/2_) is then reached by these electrons through non-radiative relaxation. After radiatively relaxing down to the ground state energy levels (^6^H_*J*/2_, where *J* = 15, 13, 11), some of these electrons release yellow (571 nm), and blue (470 nm) light. The remaining electrons undergo a process known as resonance cross-relaxation, which transfers their energy to the ^4^G_5/2_ level of Sm^3+^. The emission of yellow light (^4^G_5/2_ → 6H_5/2_), orange light (^4^G_5/2_ → ^6^H_7/2_), and red light (^4^G_5/2_ → ^6^H_9/2_) from Sm^3+^ is increased by this energy transfer.

#### 3.2.2. Polychromatic luminescence analysis

The CIE chromaticity diagram for the Ba_2−0.15−*y*_ZnSi_2_O_7_:1.5 mol% Dy^3+^, *y*Sm^3+^ (*y* = 0.2, 0.5, 1, 2, 3 mol%) phosphors under 403 nm excitation wavelengths is shown in Fig. S3. Each samples associated color temperature (CCT) was determined using McCamy's approximation,^45^

10 CCT = −449*n*^3^ + 3525*n*^2^ − 6823.3*n* + 5520.33

where *n* represents the inverse slope line and is computed as 
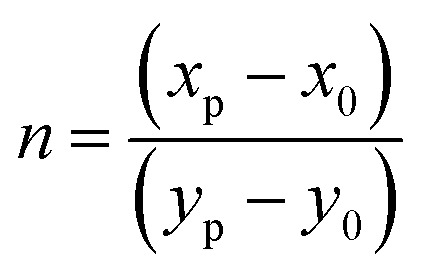
 where the chromatic coordinates of the prepared phosphor are represented by (*x*_p_, *y*_p_) and the epicenter of convergence is represented by (*x*_0_, *y*_0_) = (0.332, 0.186). The findings such as chromaticity coordinates, dominant wavelength and CCT values are shown in Table S4. The color coordinates of the Ba_2−0.15−*y*_ZnSi_2_O_7_:1.5 mol% Dy^3+^, *y*Sm^3+^ (*y* = 0.2, 0.5, 1, 2, 3 mol%) series, progressively change from yellow to orange is shown in Fig. S3. In summary, it is possible to control energy transfer and generate adjustable multicolour luminescence by adjusting the Dy^3+^/Sm^3+^ doping ratio and the excitation wavelength. The color purity was also calculated using the eqn (11),11
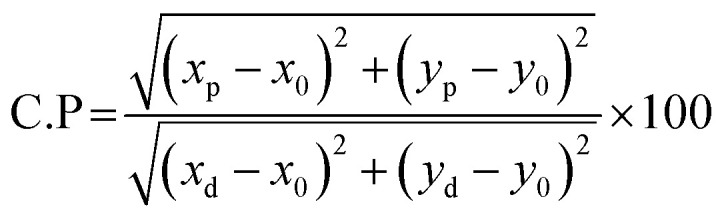
Here, (*x*_d_, *y*_d_) represent the chromaticity coordinates of the dominant wavelength. The optimized sample exhibits a colour purity of approximately 80%. As summarized in Table 3, the colour purity is observed to increases with increasing Sm^3+^ doping with Dy^3+^ ions concentration, indicating a gradual transition from pale red to bright red emission, which remains suitable for solid-state lighting applications.

**Table 3:** Band transitions, nephelauxetic ratio (*β*), and bonding parameter (*δ*) for the Ba_2_ZnSi_2_O_7_:1.5 mol% Dy^3+^, 1 mol% Sm^3+^ phosphors

Sl. no.	Transitions	*ϑ*_c_ (cm^−1^)	*ϑ*_a_ (cm^−1^)	*β*
1	^6^H_15/2_ → ^6^F_5/2_	12 341	12 432	0.9926
2	^6^H_5/2_ → ^6^F_11/2_	10 500	10 517	0.9983
3	^6^H_5/2_ → ^6^F_9/2_	9233	9136	1.0218
4	^6^H_15/2_ → ^6^F_11/2_ + ^6^H_9/2_	7637	7692	0.9928
5	^6^H_5/2_ → ^6^F_5/2_	7253	7131	1.0171
*β̄*				1.0045

### 3.3. Diffuse reflectance studies

**Fig. 7:**
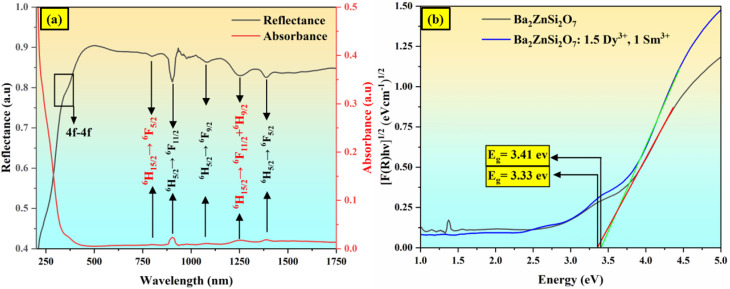
(a) Reflectance and absorbance spectra (b) Tauc plot for the Ba_2_ZnSi_2_O_7_:1.5 mol% Dy^3+^, 1 Sm^3+^ and Ba_2_ZnSi_2_O_7_.

UV-visible absorption spectroscopy is commonly used to estimate the optical band gap, which is a crucial feature for comprehending a materials optical behaviour. UV-visible spectra were obtained in diffusion reflectance mode in order to determine the optical band gap of the synthesized materials. Tauc's equation and the Kubelka–Munk function were used to estimate the band gap. The reflectance and absorbance spectra were shown in Fig. 7(a).

The absorption peaks at about 400 nm are associated with the 4f–4f transitions of RE ions. The transitions that drive the absorption peaks at higher wavelengths, including 790, 902, 1080, 1252, and 1394 nm, are driven from the ground state ^6^H_15/2_ → ^6^F_5/2_, ^6^H_5/2_ → ^6^F_11/2_, ^6^H_5/2_ → ^6^F_9/2_, ^6^H_15/2_ → ^6^F_11/2_ + ^6^H_9/2_ and ^6^H_5/2_ → ^6^F_5/2_ of Dy^3+^ and Sm^3+^ ion, respectively.^46^ In addition to it, Ba_2−*x*−*y*_ZnSi_2_O_7_:*x*Dy^3+^, *y*Sm^3+^ (*x* = 1.5, *y* = 1 mol%) has very low absorbance across the measuring range, as seen by the absorption spectra, and will thus show higher transparency in this range.^47^ The measured DRS of the material was transformed into the K–M function, which can be stated as eqn (12), in order to assess the optical energy of the phosphor.12
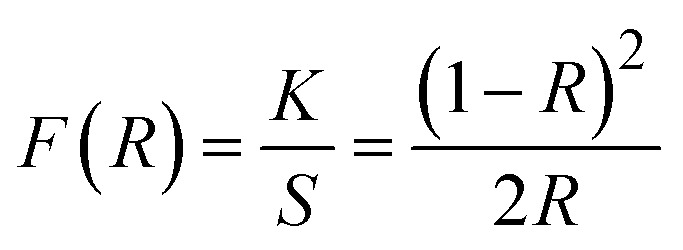
Here, *S* is the scattering coefficient, and *R* is the molar absorption, and the K–M function is represented by *F*(*R*) = *K*/*S*. Tauc's equation describes the relationship between the linear absorption coefficient (*α*) and the band gap (*E*_g_), as seen below,^48^13
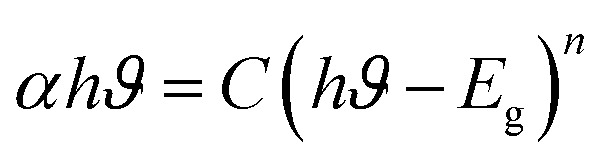


Eqn (14) derived from eqn (12) and (13), helps in calculating energy band gap,^49^14
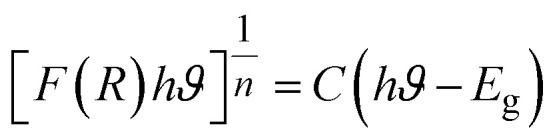
*C* is a proportionality constant in this equation, and *hϑ* is the incident photons energy. A constant variable, and *n* characterizes the electronic transitions. The value of *n* can be either 
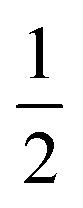
, 2
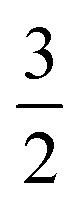
, or 3, depending on whether the transition is direct permitted, indirect allowed, direct prohibited, or indirect forbidden. We chose *n* = 
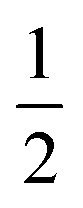
for our computations since the host matrix in our investigation has a straight band gap.^50^ By projecting the resultant curve to the *hϑ* axis as the *x*-axis, where [*F*(*R*)*hϑ*]^2^ = 0, one may find the sample's band gap (*E*_g_). As seen in the inset of Fig. 7(b), this is accomplished by plotting 
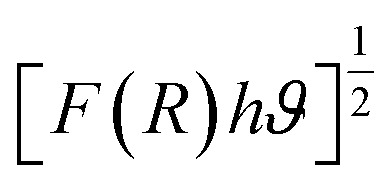
 against *hϑ*. The co-doped Ba_2_ZnSi_2_O_7_:1.5 mol% Dy^3+^, 1 Sm^3+^ and Ba_2_ZnSi_2_O_7_ have band gap values of 3.41 eV and 3.33 eV, respectively. As shown in Fig. 7(b), the negative impact outlined in the Burstein–Moss (B–M) hypothesis explains the minor increase in the optical energy gap from the host material to the co-doped sample. Using the optical band gap energy data of the prepared samples, two crucial parameters – the refractive index and the metallization criterion were computed. A difference in the energy bands is shown by the light reflected off the particles, but the light emitted from the prepared powders is known to have static qualities. A materials refractive index (*n*) is determined by the way light interacts with its atom's electrons. To calculate the refractive index values, the following eqn (15) was utilized,^51^15
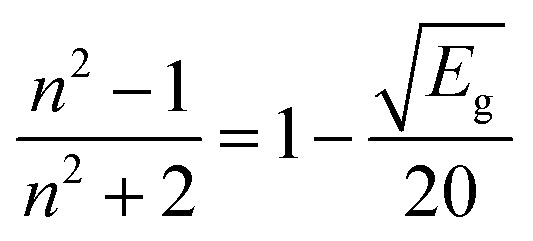
*n* is the refractive index and *E*_g_ is the band gap in the equation above. A boost in the quantity of non-bridging oxygen atoms is the main cause of the refractive index's increase from 5.35 to 5.52 brought on by the addition of Sm^3+^ ions. From the findings, refractive index values rise as the energy band gap shrinks. The metallization requirements were also examined in order to assess the insulating qualities of the produced powders in more detail. The eqn (16) that links the energy band gap, refractive index, and metallization criterion was presented by Sakka and Dimitrov and is displayed below,^52^16
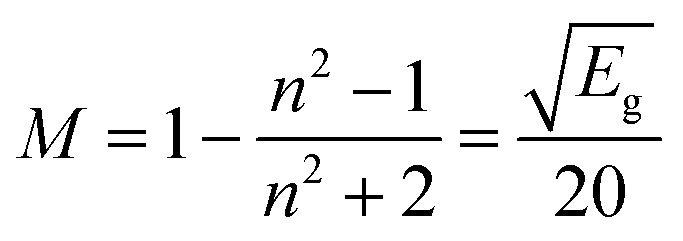


The formula put out by Sakka and Dimitrov states that metallic behaviour is suggested by a value of *M* larger than one, whereas non-metallic behaviour is indicated by a value of *M* less than one. It is clear that the produced pure and Ba_2_ZnSi_2_O_7_:1.5 mol% Dy^3+^, 1 mol% Sm^3+^ have 0.220 and 0.229 respectively so it behaves in a non-metallic manner. Understanding the nature of the Dy^3+^–Sm^3+^-ligand bond in the synthesized phosphors is aided by the calculation of the bonding parameter (*δ*) and the nephelauxetic ratio (*β*). The following is the definition of the nephelauxetic ratio,^53^17
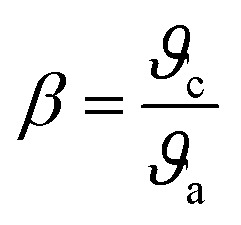
where *ϑ*_c_ and *ϑ*_a_ are the wave numbers for a specific Dy^3+^ and Sm^3+^ transition in the host and aqueous solution, respectively. Given by the bonding parameter (*δ*) is,^54^18
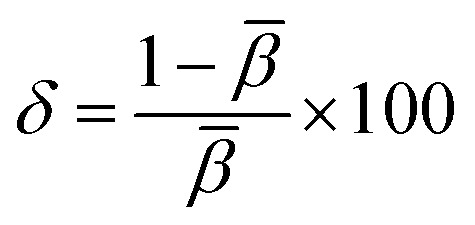


The symbol for the average nephelauxetic ratio is β*¯*. The Dy^3+^–Sm^3+^-ligand bond is categorized as covalent when *δ* is positive, and as ionic when *δ* is negative. Table 3 lists the systems band assignments, bonding parameter (*δ*), and nephelauxetic ratio (*β*). The bonding parameter is positive since *δ* = − 0.4479 is smaller than 0. This suggests the ionic character of the Dy^3+^–Sm^3+^-ligand linkages.

### 3.4. Surface morphology

**Fig. 8:**
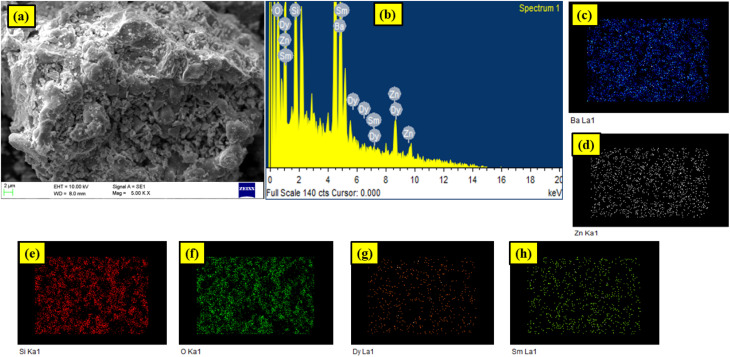
(a) SEM image (b) EDAX spectrum (c–h) elemental mapping of Ba_2_ZnSi_2_O_7_:1.5 mol% Dy^3+^, 1 mol% Sm^3+^ phosphor.

The SEM image of Ba_2_ZnSi_2_O_7_:1.5 mol% Dy^3+^, 1 mol% Sm^3+^ phosphor is shown in Fig. 8(a). It is clear from the SEM examination that the optimized sample's particles are in the micron size range and have irregular forms. The average particle size is measured from SEM found to be 1.14 µm as shown in Fig. S4. Agglomeration during heating, which produces gas byproducts, may be the cause of this.^55^ The EDX spectrum investigation has confirmed the synthesis of pure Ba_2_ZnSi_2_O_7_:1.5 mol% Dy^3+^, 1 mol% Sm^3+^ phosphor. The elements contained in the constructed lattice are represented by separate peaks in the EDX spectrum as shown in Fig. 8(b). The barium, zinc, silicon, dysprosium, samarium and oxygen peaks in the pattern show that there are no other elements present and that the intended lattice with the right stoichiometry is properly formed. The components found in the optimized sample are shown in detail in the Fig. 8(c–h). Dysprosium and samarium homogeneous incorporation into the lattice is further supported by the separate peaks for the Dy^3+^ and Sm^3+^ ion. The EDX atomic and weight percentages for the Ba_2_ZnSi_2_O_7_:1.5 mol% Dy^3+^, 1 mol% Sm^3+^ phosphor are shown in Table 4. The EDX results show that the elements are distributed as predicted and that the phosphors composition is homogenous. These results are consistent with the spectral and structural data.

**Table 4:** Elemental composition of Ba_2_ZnSi_2_O_7_:1.5 mol% Dy^3+^, 1 mol% Sm^3+^ phosphor

Element	Weight%	Atomic%
O K	20.80	58.64
Si K	9.44	14.75
Zn L	12.46	8.36
Ba L	56.00	17.89
Sm L	0.65	0.19
Dy L	1.30	0.35
Totals	100.00	100.00

### 3.5. Fourier transform IR spectra

**Fig. 9:**
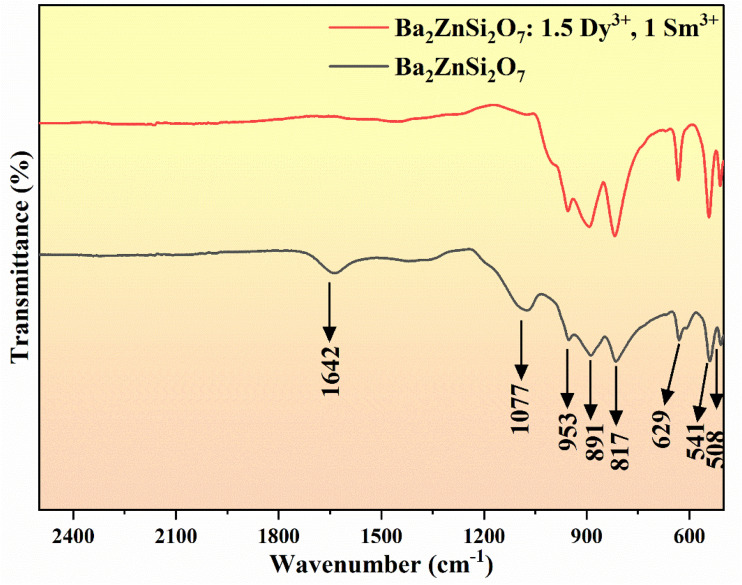
FTIR spectra of pure host and Ba_2_ZnSi_2_O_7_:*x*Dy^3+^, *y*Sm^3+^ (*x* = 1.5, *y* = 1 mol%) phosphor.

Additionally, FT-IR spectra were captured, as Fig. 9 illustrates. The findings showed that distinctive peaks associated with Si–O vibrations at wavenumbers of 508 and 891 cm^−1^ are present in both pure and Dy^3+^–Sm^3+^ co-doped samples. The Si–O–Si peak is commonly observed at wavenumbers 541 and 953 cm^−1^.^56^ Furthermore, Zn–O vibrations emerged about 817 cm^−1^, whereas Ba–O vibrations were detected around 629 cm^−1^. A peak at 1077 cm^−1^ indicates that Si–Si bonds are straining.^57^ All things considered, the FT-IR spectra indicate that the elements bonding is consistent and well-integrated which confirms structural deformation has not happened on doping. The vibrational modes corresponding to wavenumber are listed in Table 5. The vibrational modes of pure Ba_2_ZnSi_2_O_7_ and Ba_2_ZnSi_2_O_7_:1.5 Dy^3+^, 1 Sm^3+^ are found to be same which correlates that even after doping there is no change in the structural properties. This confirms that the structure is stable even after doping.

**Table 5:** Band assignments of pure host and Ba_2_ZnSi_2_O_7_:1.5 Dy^3+^, 1 Sm^3+^ phosphors

Wavenumber (cm^−1^)	Vibrational modes
508	Si–O vibrations^56^
541	Si–O–Si bending^56^
629	Ba–O vibrations^57^
817	Zn–O stretching^57^
891	Si–O vibrations^56^
953	Si–O–Si bond stretching^56^
1077	Si–Si stretching^57^

### 3.6. Thermogravimetry analysis

**Fig. 10:**
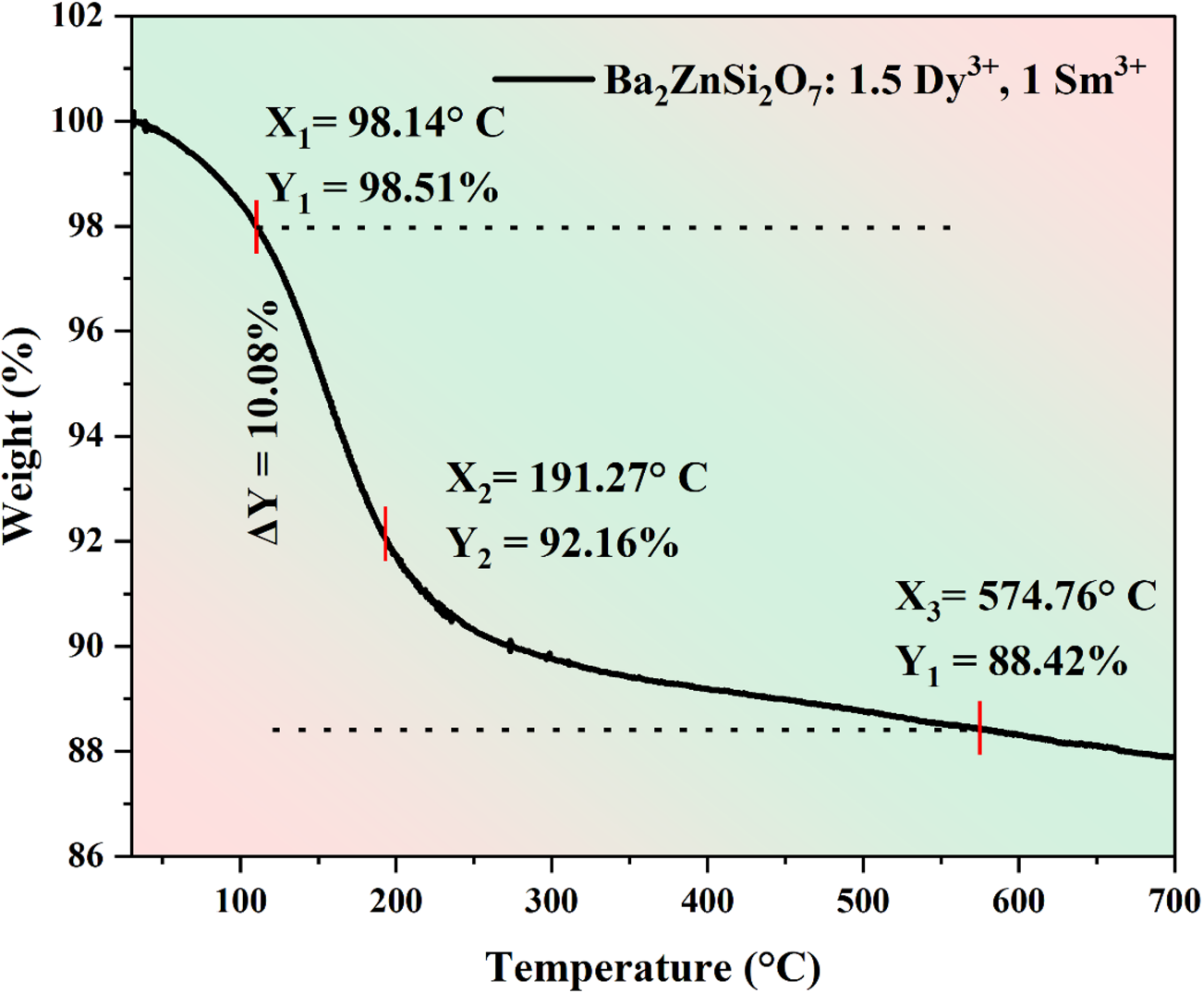
TGA curves of Ba_2_ZnSi_2_O_7_:1.5 Dy^3+^, 1 Sm^3+^ phosphor.

The TGA findings for the Ba_2_ZnSi_2_O_7_:1.5 Dy^3+^, 1 Sm^3+^ phosphor are shown in Fig. 10. As the temperature rises, the TGA curve shows the sample's weight changes. There is weight loss up to 275 °C, the TGA curve is initially decreases, showing that the material is slightly unstable in this temperature range. Following this, weight loss is shown in the first stage as shown in inset. After 275 °C material did not loss any weight reflecting it is stable at high temperature range. The loss of weight is only 8% may be due to environmental oxygen presence and hydroxyl presence. Despite the high synthesis temperature of 1200 °C, the overall weight loss of about 11% up to 600 °C can be ascribed to the removal of physically adsorbed water, surface hydroxyl groups, and trace amounts of volatile species that may have been absorbed during post-synthesis handling under ambient conditions. It is well known that powdered oxide phosphors with high surface area can easily absorb atmospheric moisture, leading to appreciable weight loss during thermal analysis. Notably, the TGA profile lacks any sharp weight loss transitions or phase-related features, signifying the absence of structural decomposition in the temperature range of interest. The fact that the weight loss is a gradual process suggests that it is dominated by desorption rather than any degradation of the oxide structure. Additionally, the observed thermal stability above 600 °C is in line with the high-temperature synthesis conditions and the known thermal stability of vanadate-based oxide phosphors.

### 3.7. Temperature dependent photoluminescence

**Fig. 11:**
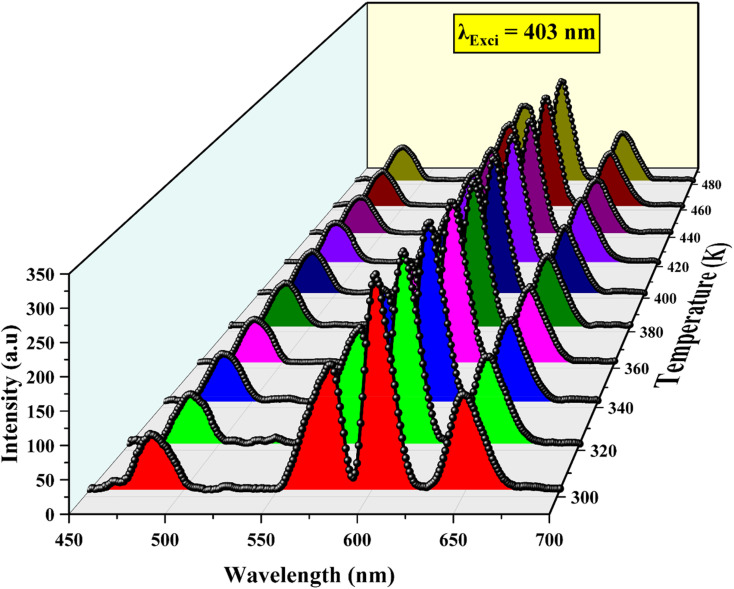
Temperature dependent photoluminescence spectra of Ba_2_ZnSi_2_O_7_:1.5 Dy^3+^, 1 Sm^3+^ phosphor.

**Fig. 12:**
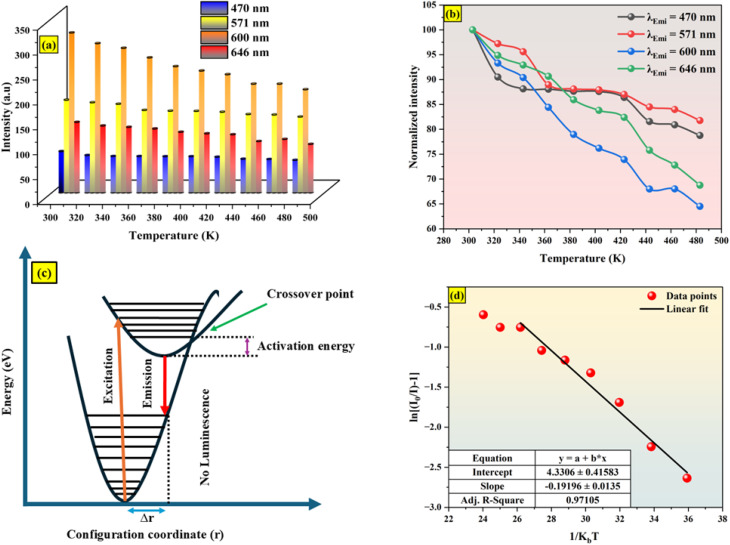
(a) Variation of intensity of different emission (b) normalized intensity of different peaks with respect to temperature (c) configurational coordinate diagram (d) Arrhenius plot for the Ba_2_ZnSi_2_O_7_:1.5 Dy^3+^, 1 Sm^3+^ phosphor.

The temperature-dependent PL spectra, which are presented in Fig. 11, were obtained at temperatures between 303 and 483 K in order to investigate the temperature sensitivity of Ba_2_ZnSi_2_O_7_:1.5 Dy^3+^, 1 Sm^3+^ phosphor as a temperature sensor. Both Dy^3+^ and Sm^3+^ emissions are progressively declining as the temperature rises from 303 to 483 K because of the thermal quenching. On the other hand, it is found that the intensity of Sm^3+^ emissions decreases considerably faster than the negligible change of Dy^3+^ emissions as shown in Fig. 12(a). Fig. 12(b) illustrates the normalized intensity variations of different emission peaks of Dy^3+^ and Sm^3+^ ions. It is observed that Dy^3+^ emission of the ^4^F_9/2_ → ^6^H_13/2_ and ^4^F_9/2_ → ^6^H_15/2_ transitions are reduced only to 22% whereas the emission from Sm^3+^ ions having ^4^G_5/2_ → ^6^H_7/2_ and ^4^G_5/2_ → ^6^H_9/2_ transitions are reduced to 36%. The reduce in the emission intensity was clearly due to thermal quenching phenomenon. The configurational coordinate diagram in Fig. 12(c) provides an explanation of the cause of the thermal quenching effect. The horizontal distance between the ground state and excited state equilibrium positions, or *i.e.*, increases as the temperature rises. As a result, before reaching the excited state's equilibrium position, where the radiative transition takes place, more electrons from the upper vibrational level of the excited state must pass the ground-excited state intersection point. The non-radiative transition rises as a result. The luminescence is considered to quench if the temperature is high enough to prevent emission and the crossover point approaches before the excited state's equilibrium configuration.^58^ This explains the reason behind emission intensity decreases as the temperature rises over 303 K. In addition to multiphoton relaxation, other non-radiative pathways may also contribute to thermal quenching. For instance, in co-doped systems like Dy^3+^/Sm^3+^, thermally activated cross-relaxation or energy transfer from Sm^3+^ to Dy^3+^ could become more probable at elevated temperatures, leading to a reduction in emission intensity. These processes are consistent with the observed decrease in fluorescence intensity and lifetime with increasing temperature. Moreover, the relatively low activation energy supports the possibility of phonon-assisted quenching pathways dominating in this temperature range.

The excellent thermal stability achieved in the present phosphor is due to the unique structure of the host Ba_2_ZnSi_2_O_7_ with Dy^3+^ and Sm^3+^.^59^ The host lattice's thermal stability may be assessed using the activation energy (*E*_A_), which can be derived from the modified Arrhenius equation as follows,19
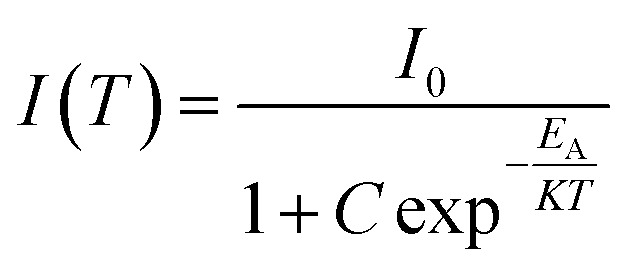
where “*K*” is the Boltzmann constant, “*C*” is a constant with the same loss, and “*I*_0_” and “*I*(*T*)” stand for the initial's intensity at room temperature and at different temperatures (*T*). Based on the slope of linear fitting of 
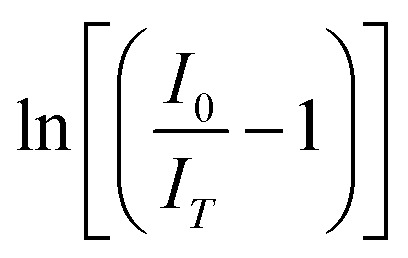
, the activation energy (*E*_A_) for the generated phosphors is 0.19 eV, and the intercept ln *C* is 4.33, as shown in Fig. 12(d). Using Boltzmann fitting to the normalized emission intensity data, the *T*_Q_ values the temperature at which phosphors lose half of their intensity values have been found,^60^20
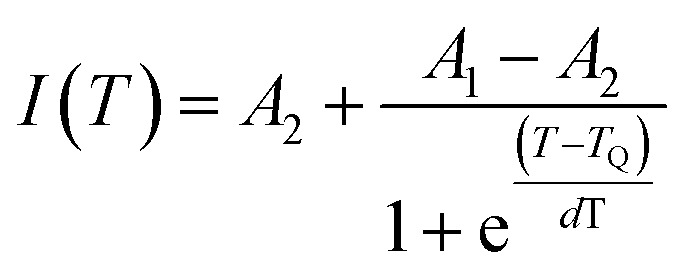


**Fig. 13:**
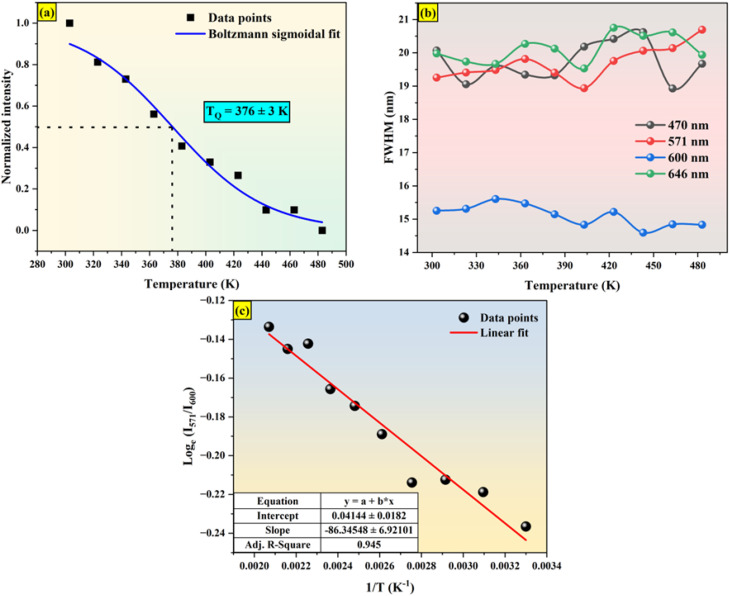
(a) Boltzmann sigmoidal fit (b) FWHM variation with respect to temperature (c) plot of log *e*(*I*_571_/*I*_600_) *versus* 1/*T*.

“*A*_1_” and “*A*_2_” represent the initial and terminal values of the curve, or the left and right horizontal asymptotes, respectively, while “*I*(*T*)” denotes the normalized emission intensity at a specific temperature in Kelvin. The fitting was conducted on the integral of the normalized emission intensity, with “*A*_1_” and “*A*_2_” assigned values of 1 and 0, respectively. In this context, the overall decrease in light production is less than the expected variation in emission intensity. Additionally, it remains unaffected by the shift in peak position that occurs during heating. “d*T*” signifies the change in “*T*” concerning the most significant alteration in “*I*(*T*)” values, and “*T*_Q_” indicates the center value of the sigmoid.^61^ This fitting yields the “*T*_Q_” value of 376 K, as seen in Fig. 13(a).

The Ba_2_ZnSi_2_O_7_:1.5 Dy^3+^, 1 Sm^3+^ phosphor's FWHM change of TDPL emission peaks with temperature is shown in Fig. 13(b). It is evident that for the 470 nm, 570 nm, 600 nm, and 646 nm emissions, the emission's full width at half maximum (FWHM) gradually narrowed. The lower interaction between the thermally stimulated luminous core and thermally active phonons is responsible for this decrease in FWHM. As the phonon population density rises at high temperatures, the electron–phonon interaction intensifies.^62^ The mathematical formula for the rise in FWHM is,21
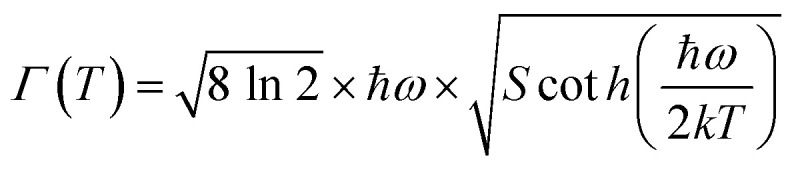
where *k* and *S* stand for the Boltzmann and Huang–Rhys parameters, respectively, *ħω* for the effective phonon energy, and *Γ*(*T*) for the temperature-dependent FWHM.^63^ The intensity ratios of anti-Stokes to Stokes peaks (*I*_a_/*I*_s_), which had a Boltzmann-type distribution function, were thought to be responsible for the variation in thermal behaviour,^64^22
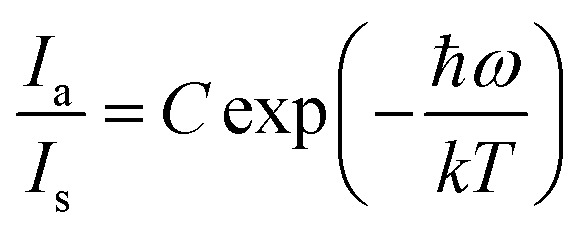


The proportionality constant is shown by *C* in this expression, whereas *T* stands for the absolute temperature. Using the difference in intensity between the anti-Stokes and Stokes emission lines is one potential method for heat sensing. A natural logarithm can be used to express eqn (22),^65^23
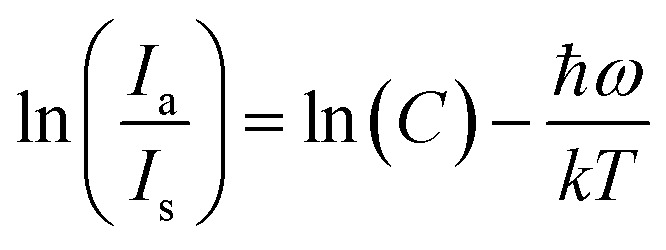


In this case, the emissions at 571 nm and 600 nm are represented by *I*_a_ and *I*_s_, respectively. The log *e*(*I*_571_/*I*_600_) *versus* 1/*T* which is shown in Fig. 13(c). The data was fitted linearly, and the best fit was found with *ħω*/*k* = 86.34 K from the relationship 
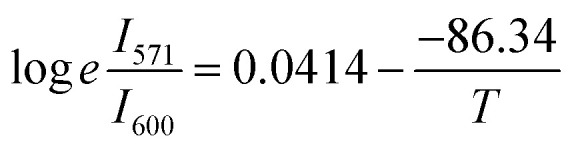
. It was discovered that the phonon energy (*ħω*) was 60.009 cm^−1^. These findings imply that the improved phosphor's reduced phonon energy may enable efficient temperature detection.^66^

### 3.8. Thermal sensing studies

#### 3.8.1. Fluorescence intensity ratio (FIR method)

**Fig. 14:**
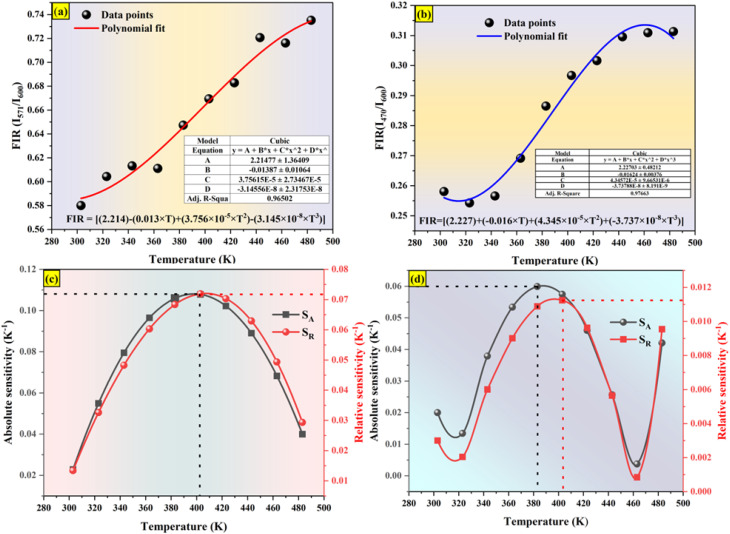
Fluorescence intensity ratio of (a) *I*_571_/*I*_600_ (b) *I*_470_/*I*_600_ (c) relative sensitivity and absolute sensitivity of *I*_571_/*I*_600_ (d) relative sensitivity and absolute sensitivity of *I*_470_/*I*_600_.

The temperature dependence of integrated intensities of Sm^3+^: ^4^G_5/2_ → ^6^H_7/2_ with Dy^3+^: ^4^F_9/2_ → ^6^H_13/2_ and ^4^F_9/2_ → ^6^H_13/2_ are displayed in Fig. 11, respectively, to examine the sensing properties. It is evident that, in contrast to Dy^3+^, the integrated intensity of Sm^3+^ varied quickly. The FIR_1_ is considered for *I*_571_/*I*_600_ and FIR_2_ is for *I*_470_/*I*_600_ as shown in Fig. 14(a and b). FIR is distinguished by significant temperature dependency and strong sensitivity to temperature changes, and it functions irrespective of the excitation source or luminous center concentration. The relative population of the thermally related energy levels frequently follows the Boltzmann distribution. The effects of temperature on emission intensities due to the Boltzmann distribution of thermally linked energy levels. It is possible to represent the correlation between temperature and the excited rare earth ions PL intensity as shown by polynomial function,^67^24
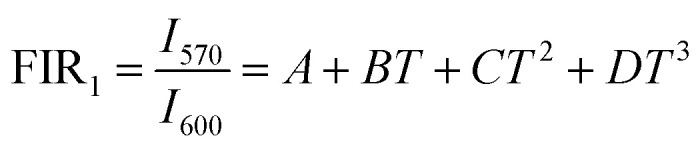
25
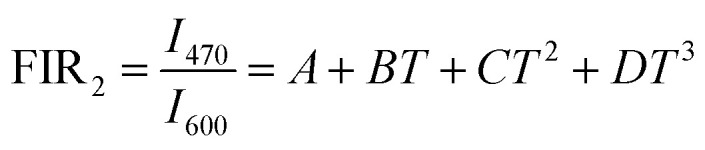
where *A*, *B*, *C*, and *D* are the fitting constants for the polynomial. The sensitivity might be determined using this equation by taking the first derivative of the FIR. In Fig. 14(a and b), the polynomial equation is fitted for both FIR_1_ and FIR_2_ is displayed. A best fit for the FIR data points is provided by FIR_1_ = [(2.214) − (0.013 × *T*) + (3.756 × 10^−5^ × *T*^2^) − (3.145 × 10^−8^ × *T*^3^)] and FIR_2_ = [(2.227) + (−0.016 × *T*) + (4.345 × 10^−5^ × *T*^2^) + (−3.737 × 10^−8^ × *T*^3^)], respectively, as seen in Fig. 14(a and b). Sensitivity is the most crucial feature of a temperature sensor to gain a better understanding of the material is used and its performance. Relative temperature sensitivity (*S*_R_) is another crucial element for sensing applications. A large variety of temperature sensor types are compared by *S*_R_.^68,69^ The following equations calculate and illustrate the absolute sensitivity (*S*_A_) and relative sensitivity (*S*_R_) of the third-order polynomial functions.^70–72^26
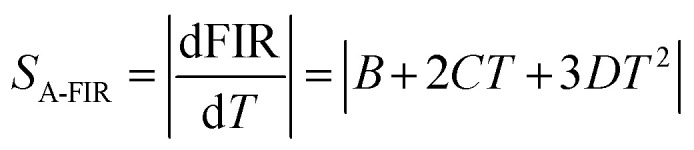
27
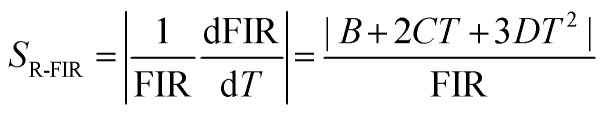


Fig. 14 (c) and (d) displays the measured absolute and relative sensitivity levels at various temperatures. For FIR_1_, *S*_R-FIR_ is found to be 7.19% K^−1^ and *S*_A-FIR_ to be 0.1086 K^−1^ at 403 K and for FIR_2_, *S*_R-FIR_ found to be 1.12% K^−1^ at 403 K and *S*_A-FIR_ calculated to be 0.0599 K^−1^ at 383 K, when FIR is computed using the third order polynomial function. Table 6 lists a few popular bright thermometers based on Dy^3+^ and Sm^3+^ doped/co-doped phosphors that have been previously reported. It is demonstrated that the Ba_2_ZnSi_2_O_7_:1.5 Dy^3+^, 1 Sm^3+^ phosphor has a rather high *S*_R_ value. Consequently, the Ba_2_ZnSi_2_O_7_:1.5 Dy^3+^, 1 Sm^3+^ phosphor may be employed in optical thermometry and shows excellent optical thermometric performance.

**Table 6:** With an emphasis on *S*_R_ and their *S*_A_ temperature sensitivity, a comparison of temperature sensing materials

Materials	Temperature range (K)	*S*_R-max_ (% K^−1^)	Mode	Ref.
K_3_YF_6_:Dy^3+^/Sm^3+^ (GC)	298–448	0.401	FIR	77
Sr_2_Y_8_(SiO_4_)_6_O_2_:Sm^3+^	100–500	1.11	FIR	78
		0.94	FLT	
Y_2_MgTiO_6_:Sm^3+^	298–498	0.26	FIR	79
CaWO_4_:Dy^3+^	302–650	2.77	FIR	80
SrMoO_4_:Dy^3+^	303–483	0.39	FIR	81
Li_2_TiO_3_/Y_2_O_3_:Dy^3+^	273–373	6.67	FLT	82
BaLaMgNbO_6_:Dy^3+^, Mn^4+^	230–470	2.43	FLT	83
BaGd_2_O_4_:Bi^3+^/Sm^3+^	293–473	1.66	FLT	84
Ba_2_ZnSi_2_O_7_:1.5 Dy^3+^	303–483	2.05	FIR	27
		5.42	FLT	
Ba_2_ZnSi_2_O_7_:1.5 Dy^3+^, 1 Sm^3+^	303–483	7.19	FIR	This work
		1.10	FLT	

#### 3.8.2. Fluorescence lifetime method (FLT)

Instrument uncertainty, phosphor degradation, and other problems may limit the accuracy of mono-mode optical temperature sensors that employ FIR technology. The precision of temperature readings may be improved in optical temperature sensing by using dual-mode FIR and various luminescence lifetimes. The fluorescence lifetime of Sm^3+^ in the instance of Ba_2_ZnSi_2_O_7_:1.5 Dy^3+^, 1 Sm^3+^ falls dramatically as the temperature rises from 303 K to 483 K (*λ*_ex_ = 403 nm, *λ*_em_ = 600 nm). In Ba_2_ZnSi_2_O_7_:1.5 Dy^3+^, 1 Sm^3+^, the Sm^3+^ luminescence lifetime is 1.9714 ms at 303 K and increases to 2.0239 ms at 483 K. These findings imply that it can be used as a luminous thermometer that lasts for long period of time. The following formula can be used to determine the activation energy for Ba_2_ZnSi_2_O_7_:1.5 Dy^3+^, 1 Sm^3+^ thermal quenching,^73,74^28
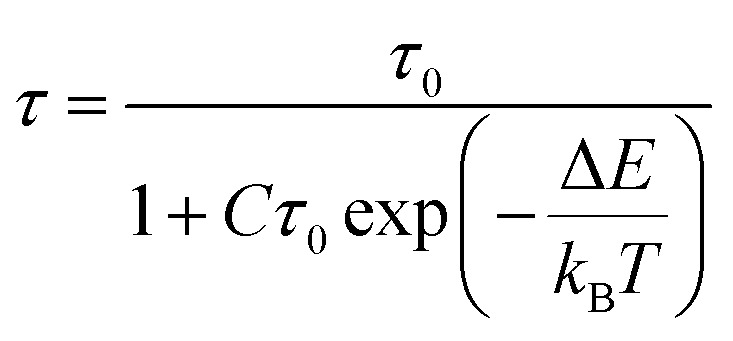


**Fig. 15:**
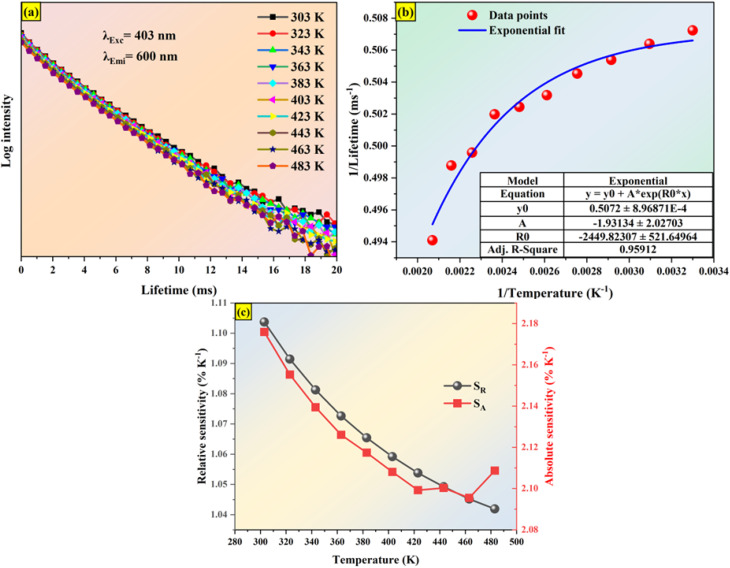
(a) Single exponential fitted lifetime spectra for different temperature (b) fluorescence lifetime fit (c) relative sensitivity and absolute sensitivity of lifetime-based method.

In this case, *C* is a constant, *τ*_0_ is the decay lifetime at the reference temperature, and *τ* is the decay lifetime at the current temperature *T*. Fig. 15(a and b) displays the decay lifetime curves and the exponential fitted data for the lifetime measurements. Based on their lifespan, the absolute (*S*_A-lifetime_) and relative (*S*_R-lifetime_) sensitivities of optical thermometric materials may be determined using the following formulas,^75,76^29
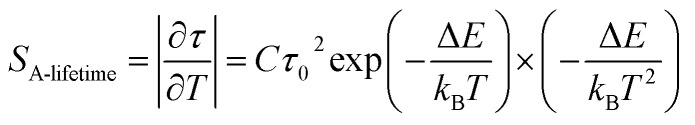
30
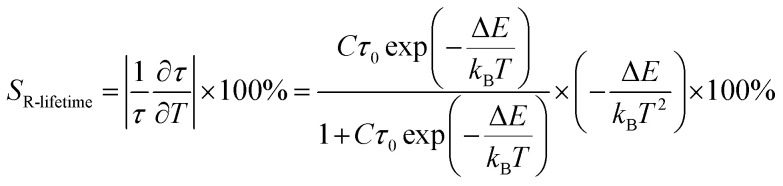


The study's findings on the differences in *S*_A-lifetime_ and *S*_R-lifetime_ are shown in Fig. 15(c). Both *S*_A-lifetime_ and *S*_R-lifetime_ decrease with temperature. At 303 K, *S*_A-lifetime_ achieves its greatest value of 0.0217 K^−1^, and *S*_R-lifetime_ reaches its maximum value of 1.10% K^−1^. Based on lifetime measurements. Table 6 displays the *S*_R-lifetime_ values for a number of phosphors that have been documented in the literature. This comparison makes it clear that the Ba_2_ZnSi_2_O_7_:1.5 Dy^3+^, 1 Sm^3+^ phosphor has a great deal of promise as a material for temperature sensing.

## 4. Conclusion

In summary, the high-temperature solid-state reaction approach was effectively used to develop and manufacture the new Ba_2_ZnSi_2_O_7_:Dy^3+^, Sm^3+^ phosphor with dual activators luminescence. For a possible use as a dual-mode optical temperature sensor, the temperature-dependent luminous characteristics of the Ba_2_ZnSi_2_O_7_:Dy^3+^, Sm^3+^ sample were examined. XRD confirmed the monoclinic structure with high phase purity. The optimum concentration of Sm^3+^ in Ba_2_ZnSi_2_O_7_:Dy^3+^ was found to be 1 mol% through photoluminescence spectroscopy. Energy transfer efficiency was calculated, and the energy transfer was observed from Dy^3+^ to Sm^3+^. The DRS spectra was studied to obtain the energy bandgap of the material which was found to be increased due to shift in the conduction band. The SEM shows agglomerated structure due to synthesis method. The FTIR spectra revealed unaltered structure even after co-doping. Under 403 nm illumination, the thermal-quenching activation energy of Sm^3+^ was determined to be 0.19 eV, the relative sensitivity peaked at 7.19% K^−1^ at 403 K, and the change of FIR (*I*_Dy_/*I*_Sm_) with temperature fluctuation is dramatic from 303 K to 483 K. Furthermore, Sm^3+^ fluorescence lifetime can also be used to sense temperature, and at 303 K, the relative sensitivity reaches a high of 1.10% K^−1^. The Ba_2_ZnSi_2_O_7_:Dy^3+^, Sm^3+^ phosphor was shown in all of these studies to be a potential option for high-sensitivity optical temperature sensors.

## Author contributions

Tejas: methodology, formal analysis, validation, investigation, data curation, writing – original draft, writing – review & editing. A. Princy: validation, software, resources, investigation. S. Masilla Moses Kennedy: visualization, software, resources. Sudha D. Kamath: writing – review & editing, visualization, validation, supervision, resources, project administration, investigation, formal analysis, data curation.

## Conflicts of interest

The authors declare that they have no known competing financial interests or personal relationships that could have appeared to influence the work reported in this paper.

## Supplementary Material

RA-016-D5RA09381C-s001

## Data Availability

The data that support the findings of this study are available from the corresponding author upon reasonable request. Supplementary information (SI): experimental procedures, supplementary figures (Fig. S1–S4), and tables (Tables S1–S4). See DOI: https://doi.org/10.1039/d5ra09381c.

## References

[cit1] Zaldo C. , Lanthanide-based luminescent thermosensors: From bulk to nanoscale, Lanthanide-Based Multifunctional Materials: from OLEDs to SIMs, Elsevier, 2018, pp. 335–379, 10.1016/B978-0-12-813840-3.00010-7

[cit2] Dramićanin M. D. (2016). Sensing temperature via downshifting emissions of lanthanide-doped metal oxides and salts. A review. Methods Appl. Fluoresc..

[cit3] Sójka M., Piotrowski W., Marciniak L., Zych E. (2024). Co-doping to extend the operating range of luminescence thermometers. The case of Y2SiO5:Pr3+,Tb3+. J. Alloys Compd..

[cit4] Liu W., Yang B. (2007). Thermography techniques for integrated circuits and semiconductor devices. Sens. Rev..

[cit5] McLaurin E. J., Bradshaw L. R., Gamelin D. R. (2013). Dual-emitting nanoscale temperature sensors. Chem. Mater..

[cit6] Wang X. D., Wolfbeis O. S., Meier R. J. (2013). Luminescent probes and sensors for temperature. Chem. Soc. Rev..

[cit7] Dramićanin M. D. (2020). Trends in luminescence thermometry. J. Appl. Phys..

[cit8] Kobylinska A., Kniec K., Maciejewska K., Marciniak L. (2019). The influence of dopant concentration and grain size on the ability for temperature sensing using nanocrystalline MgAl2O4:Co2+,Nd3+ luminescent thermometers. New J. Chem..

[cit9] Brites C. D. S. , Millán A. and Carlos L. D., Lanthanides in Luminescent Thermometry, Handbook on the Physics and Chemistry of Rare Earths, Elsevier B.V., 2016, vol. 49, pp. 339–427, 10.1016/bs.hpcre.2016.03.005

[cit10] Brites C. D. S., Balabhadra S., Carlos L. D. (2019). Lanthanide-Based Thermometers: At the Cutting-Edge of Luminescence Thermometry. Adv. Opt. Mater..

[cit11] Bednarkiewicz A., Drabik J., Trejgis K., Jaque D., Ximendes E., Marciniak L. (2021). Luminescence based temperature bio-imaging: Status, challenges, and perspectives. Appl. Phys. Rev..

[cit12] Jaque D., Vetrone F. (2012). Luminescence nanothermometry. Nanoscale.

[cit13] C Lal S., I N. J., Ganesanpotti S. (2023). A six-mode optical thermometry rooted from the distinct thermal behavior of SrLaLiTeO6: Mn4+, Eu3+ double perovskites and their potential application in wavelength detection. J. Sci.:Adv. Mater. Devices.

[cit14] Blasse G. , Grabmaier B. C., Blasse G. and Grabmaier B. C., A General Introduction to Luminescent Materials, Springer, Berlin, Heidelberg, 1994, pp. 1–9

[cit15] Thermographic Phosphors Sami Alaruri T. D. , Brewington A. J., Thomas M. A. and Miller J. A., High-Temperature Remote Thermometry Using Laser-Induced Fluorescence Decay Lifetime Measurements of Y203, 1993, vol. 42

[cit16] Noel B. W. , Turley W. D. and Allison S. W., Thermographic-phosphor Temperature Measurements: Commercial and Defense-Related Applications (No. EGG-11265-2053; CONF-940581-2), EG and G Energy Measurements, Inc., Goleta, CA (United States), 1994

[cit17] Heyes A. L., Seefeldt S., Feist J. P. (2006). Two-colour phosphor thermometry for surface temperature measurement. Opt. Laser Technol..

[cit18] Särner G., Richter M., Aldén M. (2008). Investigations of blue emitting phosphors for thermometry. Meas. Sci. Technol..

[cit19] Nakajima T. , Utsunomiya M. and Ikeda Y., Simultaneous Measurement of Velocity and Temperature of Water Using LDV and Fluorescence Technique, n.d

[cit20] Khalid A. H., Kontis K. (2009). 2D surface thermal imaging using rise-time analysis from laser-induced luminescence phosphor thermometry. Meas. Sci. Technol..

[cit21] Dosev D., Kennedy I. M., Godlewski M., Gryczynski I., Tomsia K., Goldys E. M. (2006). Fluorescence upconversion in Sm-doped Gd 2O 3. Appl. Phys. Lett..

[cit22] Treadaway M. J., Powell R. C. (1975). Energy transfer in samarium-doped calcium tungstate crystals. Phys. Rev. B.

[cit23] Yao S., Li Y., Xue L., Yan Y. (2011). Synthesis and luminescent properties of a novel red-emitting phosphor Ba2ZnSi2O7:Eu3+, B3+ for ultraviolet light-emitting diodes. Int. J. Appl. Ceram. Technol..

[cit24] Yao S., Li Y., Xue L., Yan Y. (2010). Photoluminescence properties of Ba2ZnSi2O7:Eu2+, Re3+ (Re = Dy, Nd) long lasting phosphors prepared by the combustion-assisted synthesis method. J. Alloys Compd..

[cit25] Deng S., Qiu Z., Zhang M., Zhou W., Zhang J., Li C. (2015). *et al.*, Tricolor emitting and energy transfer in the phosphor Ba2ZnSi2O7:Ce3+,Eu3+,Eu2+ for white-LED based near-UV chips. J. Rare Earths.

[cit26] Yang Z., Hu Y., Chen L., Wang X. (2014). Color tuning of Ba2ZnSi2O7:Ce 3+, Tb3+ phosphor via energy transfer. J. Lumin..

[cit27] Tejas A. P., Masilla Moses Kennedy S., Panda K., Sayyed M. I., Hanafy T. A. (2025). *et al.*, A Comprehensive Study of Nuanced Properties of Dy ^3+^ -Doped Alkaline Earth Silicates for Noncontact Thermometry Applications. Luminescence.

[cit28] Patle Y., Brahme N., Bisen D. P., Richhariya T., Chandrawanshi E., Choubey A. (2021). *et al.*, Study of Photoluminescence, Thermoluminescence, and Afterglow properties of Dy3+ doped Ba2ZnSi2O7 phosphor. Optik.

[cit29] Panda R., Behera M., Kumar R. A., Joshi D., Padhi R. K. (2023). Luminescence studies of high color purity red-emitting CaAl4O7:Eu3+ phosphor prepared by microwave-assisted synthesis technique. J. Alloys Compd..

[cit30] Banjare G. R., Bisen D. P., Brahme N., Belodhiya C. (2024). Spectroscopic investigation by incorporation of charge compensator ions in CaBaSiO4: Dy3+ phosphors for solid-state lighting applications. Indian J. Phys..

[cit31] Tejas C., Princy A., Kennedy S. M. M., Mishra V., Sayyed M. I., Hanafy T. A. (2024). Structural, thermal, and optical spectroscopic studies of Sm3+-doped Ba2ZnSi2O7 phosphors for optical thermometry applications. Mater. Adv..

[cit32] Charak I., Manhas M., Bedyal A. K., Vij A., Swart H. C., Kumar V. (2023). Synthesis, luminescence and photometric investigation of Sr2B2O5:Dy3+ phosphor for UV-based white LEDs. Appl. Phys. A:Mater. Sci. Process..

[cit33] Upadhyay K., Thomas S., Tharayil A., Tamrakar R. K. (2023). Green emitting dysprosium-activated SrY2O4 phosphor for tricolour white light-emitting diode application: structural analysis and luminescence behaviour. Chem. Pap..

[cit34] Navya N., Krushna B. R. R., Sharma S. C., Vaithy K. A., George A., Mohapatra S. S. (2024). *et al.*, A highly thermal-stable orange red emitting La(OH)_3_:Sm^3+^ phosphor for w-LED and thermal sensor dual-applications. Mater. Res. Bull..

[cit35] Ouertani G., Maciejewska K., Piotrowski W., Horchani-Naifer K., Marciniak L., Ferhi M. (2024). High thermal stability of warm white emitting single phase GdPO4: Dy3+/Sm3+ phosphor for UV excited wLEDs. J. Lumin..

[cit36] Tang H., Qin Y., Zhao X., Liu L., Huang Z., Quan J. (2024). *et al.*, Highly thermostable and color tunable Dy3+/Sm3+ co-doped germanate phosphors for solid-state lighting. J. Alloys Compd..

[cit37] Du J., Lyu S., Wang P., Wang T., Lin H. (2023). Multimode-Responsive Luminescence Smart Platform by Single-Sm3+-Doped Phosphors. Adv. Opt. Mater..

[cit38] Souadi G., Amri N., Kaynar Ü. H., Coban M. B., Madkhali O., Ayvacikli M. (2024). Novel Sm3+ doped YCa4O(BO3)3 phosphors: Structural and, low and room temperature luminescent insights. Appl. Radiat. Isot..

[cit39] Dexter T. C. , Of Pearl River, New York, Assignor to the Dexter Folder Company of New York, N. Y, Reissued Letters Patent No. 11723, dated March 14, 1899

[cit40] Blasse G. P. R. L. (1968). Energy transfer in oxidic phosphors. Phys. Lett. A.

[cit41] Van Uitert L. G. (1967). Characterization of energy transfer interactions between rare earth ions. J. Electrochem. Soc..

[cit42] Li K., Van Deun R. (2020). Color Tuning from Greenish-Yellow to Orange-Red in Thermal-Stable KBaY(MoO4)3:Dy3+, Eu3+Phosphors via Energy Transfer for UV W-LEDs. ACS Appl. Electron. Mater..

[cit43] Dai X., Zou X., Wei M., Zhang X., Dong B., Li X. (2024). Efficient and Thermally Stable Cr3+-Doped Phosphor Achieved by Cation Substitution: Plant Lighting Application. Adv. Opt. Mater..

[cit44] Luwang M. N., Ningthoujam R. S., Srivastava S. K., Vatsa R. K. (2011). Preparation of white light emitting YVO4: Ln3+ and silica-coated YVO4:Ln3+ (Ln3+ = Eu 3+, Dy3+, Tm3+) nanoparticles by CTAB/n-butanol/hexane/water microemulsion route: Energy transfer and site symmetry studies. J. Mater. Chem..

[cit45] McCamy C. S. (1992). Correlated color temperature as an explicit function of chromaticity coordinates. Color Res. Appl..

[cit46] Li X., Wang X., Li X., Cheng L., Tong L., Wang W. (2016). *et al.*, Luminescence studies of Sm3+ single-doped and Sm3+, Dy3+ co-doped NaGdTiO4 phosphors. Phys. B.

[cit47] Yang L., Mi X., Zhang H., Zhang X., Bai Z., Lin J. (2019). Tunable luminescence and energy transfer properties in Ca2NaMg2V3O12: Ln3+ (Dy3+, Sm3+) phosphors. J. Alloys Compd..

[cit48] Kumari C., Manam J., Sharma S. K. (2023). Strong red emission in double perovskite Sr3LiSbO6: Eu3+ phosphor with high color purity for solid-state lighting applications. Mater. Sci. Semicond. Process..

[cit49] Luo M., Sha X., Chen B., Zhang X., Yu H., Li X. (2022). *et al.*, Optical transition properties, internal quantum efficiencies, and temperature sensing of Er3+ doped BaGd2O4 phosphor with low maximum phonon energy. J. Am. Ceram. Soc..

[cit50] Gopal R., Kumar A., Manam J. (2021). Enhanced photoluminescence and abnormal temperature dependent photoluminescence property of SrWO4:Dy3+ phosphor by the incorporation of Li+ ion. Mater. Chem. Phys..

[cit51] Sushma K. C., Basavaraj R. B., Aarti D. P., Reddy M. B. M., Nagaraju G., Rudresha M. S. (2023). Efficient red-emitting SrZrO3:Eu3+ phosphor superstructures for display device applications. J. Mol. Struct..

[cit52] Dimitrov V., Sakka S. (1996). Linear and nonlinear optical properties of simple oxides. II. J. Appl. Phys..

[cit53] Jin Y., Liu X., Du Y., Yan K., Han W., Liu G. (2025). Novel K3Nb2F11O:Mn4+ oxyfluoride red phosphor with high performance enhanced by charge compensation strategy and its applications. Mater. Today Chem..

[cit54] Raji R., Anjana P. S., Gopakumar N. (2023). An insight into Judd-Ofelt analysis and non-contact optical thermometry of LiCa2Mg2V3O12: Dy3+ phosphors for multifunctional applications. Opt. Mater..

[cit55] Kumar P., Singh S., Gupta I., Kumar V., Singh D. (2022). Er3+ activated LaAlO3 perovskite phosphor: Crystal structure and down conversion photoluminescent behaviour for optoelectronic devices. Inorg. Chem. Commun..

[cit56] Min X., Sun Y., Kong L., Guan M., Fang M., Liu Y. (2018). *et al.*, Novel pyrochlore-type La2Zr2O7: Eu3+ red phosphors: Synthesis, structural, luminescence properties and theoretical calculation. Dyes Pigm..

[cit57] Gupta I., Singh D., Kumar P., Singh S., Bhagwan S., Kumar V. (2023). Structural, morphological, and optical characteristics of Gd2Si2O7:Dy3+ nanophosphors for WLEDs. Luminescence.

[cit58] Blasse G. , Chemistry and physics of R-activated phosphors, Handbook on the Physics and Chemistry of Rare Earths, 1979, vol. 4, pp. 237–274

[cit59] Wang G., Li J., Xu L., Yuan H., Sun X. (2024). A potential cyan phosphor for full spectrum light-emitting diodes: Bi3+ activated SrBaGdGaO5 phosphor. J. Mol. Struct..

[cit60] Zhao M., Ge Y., Li Y., Song X., Zhang X. (2024). Achieving Eu2+ Luminescence at Trivalent Lattice Site in Rb3Y(PO4)2:Eu toward Multicolor Emissions by Carbon and Hydrogen Coreduction. Adv. Funct. Mater..

[cit61] Zhang M., Dang P., Zeng Z., Liu D., Wan Y., Wei Y. (2024). Low-Coordination Crystallographic Lattice Engineering to Discover Ce3+-Activated Ultra-Wide Visible-to-Near-Infrared Luminescent Materials. Adv. Opt. Mater..

[cit62] Li L., Yang H., Wang Y., Ling F., Zhou X., Xiang G. (2024). *et al.*, Multifunctional applications of novel near-infrared emitting Ca2ScNbO6:Cr3+ double-perovskite phosphors. Ceram. Int..

[cit63] Yang S., Wang Y., Xiang G., Jiang S., Li L., Ling F. (2023). Luminescence properties and phase transformation of broadband NIR emitting A2(WO4)3:Cr3+ (A=Al3+, Sc3+) phosphors toward NIR spectroscopy applications. J. Lumin..

[cit64] Cai H., Liu S., Song Z., Liu Q. (2021). Tuning luminescence from NIR-I to NIR-II in Cr3+-doped olivine phosphors for nondestructive analysis. J. Mater. Chem. C.

[cit65] Tomar S., Mishra N. K., Kesarwani V., Rai V. K., Kumar K., Shivakumara C. (2024). Dual-Mode Light Emission and Dynamic Studies of Er3+/Yb3+-Doped NaLa(MoO4)2 Phosphor for Optical Thermometry Operating from Cryogenic to above Room Temperatures. ACS Appl. Opt. Mater..

[cit66] Barthel J., Zeiger P. M., Rusz J., Allen L. J. (2024). Simple model for phonon spectroscopy using fast electrons. Phys. Rev. B.

[cit67] Kong J., Shang X., Zheng W., Chen X., Tu D., Wang M. (2020). *et al.*, Revisiting the Luminescence Decay Kinetics of Energy Transfer Upconversion. J. Phys. Chem. Lett..

[cit68] Fan H., Lu Z., Meng Y., Chen P., Zhou L., Zhao J. (2022). Optical temperature sensor with superior sensitivity based on Ca2LaSbO6: Mn4+, Eu3+ phosphor. Opt. Laser Technol..

[cit69] Wang X., Liu Q., Bu Y., Liu C. S., Liu T., Yan X. (2015). Optical temperature sensing of rare-earth ion doped phosphors. RSC Adv..

[cit70] Huang X., Huang K., Chen L., Chen N., Lei R., Zhao S. (2020). Effect of Li+/Mg2+ co-doping and optical temperature sensing behavior in Y2Ti2O7: Er3+/Yb3+ upconverting phosphors. Opt. Mater..

[cit71] Cheng X., Dong X., Peng K., Zhang H., Su Y., Jiang L. (2020). Upconversion Luminescence and Optical Temperature-Sensing Properties of LaNbO4:Yb3+/Er3+ Phosphors. J. Electron. Mater..

[cit72] Porosnicu I., Colbea C., Baiasu F., Lungu M., Istrate M. C., Avram D. (2020). A sensitive near infrared to near-infrared luminescence nanothermometer based on triple doped Ln -Y2O3. Methods Appl. Fluoresc..

[cit73] Wang Z., Jiao H., Fu Z. (2018). Investigating the Luminescence Behaviors and Temperature Sensing Properties of Rare-Earth-Doped Ba2In2O5 Phosphors. Inorg. Chem..

[cit74] Dramićanin M. D., Milićević B., Đorđević V., Ristić Z., Zhou J., Milivojević D. (2019). *et al.*, Li2TiO3:Mn4+ Deep-Red Phosphor for the Lifetime-Based Luminescence Thermometry. ChemistrySelect.

[cit75] Çabuk S., Mamedov A. (1999). Urbach rule and optical properties of the LiNbO_3_ and LiTaO_3_. J. Opt. A: Pure Appl. Opt..

[cit76] Luo H., Li X., Wang X., Peng M. (2020). Highly thermal-sensitive robust LaTiSbO6:Mn4+ with a single-band emission and its topological architecture for single/dual-mode optical thermometry. Chem. Eng. J..

[cit77] Ke L., Cai X., Ren K., Zhang Y. (2024). Color tunability and temperature sensing capabilities in Dy3+/Sm3+ co-doped transparent oxyfluoride glass ceramics containing K3YF6 nanocrystals. Ceram. Int..

[cit78] Priya A. S., Ramachandran S., Vasantha S. D., Kumar H. P. (2024). Delineating Dy3+ and Sm3+ in thermally stable strontium silicate apatites for multifunctional applications. J. Mol. Struct..

[cit79] Long J., Yang C., Li B., Ma R., Huang W. (2024). Novel orange-red emitting phosphor Y2MgTiO6:Sm3+ luminescence properties and optical thermometry. Ceram. Int..

[cit80] Liao Z., Cao B., Li L., Cong Y., He Y., Dong B. (2023). Exploring the excitation spectrum behavior of Dy in CaWO4 for a new excited-state-based ratiometric thermometry. Appl. Mater. Today.

[cit81] Chauhan V., Dixit P., Pandey P. K., Chaturvedi S., Pandey P. C. (2023). Dy3+-Assisted Negative-Thermal Quenching in Ho3+-Doped SrMoO4 for Luminescence Thermometry and Lighting Applications. J. Phys. Chem. C.

[cit82] Xie C., Wang P., Lin Y., Wei X., Yin M., Chen Y. (2020). Temperature-dependent luminescence of a phosphor mixture of Li2TiO3: Mn4+ and Y2O3: Dy3+ for dual-mode optical thermometry. J. Alloys Compd..

[cit83] Lin Y., Zhao L., Jiang B., Mao J., Chi F., Wang P. (2019). Temperature-dependent luminescence of BaLaMgNbO6:Mn4+, Dy3+ phosphor for dual-mode optical thermometry. Opt. Mater..

[cit84] Fu J., Zhou L., Chen Y., Lin J., Ye R., Deng D. (2022). Dual-mode optical thermometry based on Bi3+/Sm3+ co-activated BaGd2O4 phosphor with tunable sensitivity. J. Alloys Compd..

