# Supplementary material for: Dual-mode optical temperature sensing using Dy3+/Sm3+ co-activated Ba2ZnSi2O7 phosphor with tuneable sensitivity
Source: RSC Adv. 2026 Feb 16;16(10):9180–200. doi: 10.1039/d5ra09381c (PMC12908135; doi:10.1039/d5ra09381c)
Supplement: RA-016-D5RA09381C-s001 [file RA-016-D5RA09381C-s001.pdf]

Supplementary Information (SI) for RSC Advances.
This journal is © The Royal Society of Chemistry 2026

Dual-mode Optical Temperature Sensing Using Dy^{3+}/Sm^{3+} Co-activated $Ba_2ZnSi_2O_7$ Phosphor with Tuneable Sensitivity

Tejas [a], A Princy [b], S Masilla Moses Kennedy [b], Sudha D. Kamath [a], *

[a] *Department of Physics, Manipal Institute of Technology, Manipal Academy of Higher Education, Manipal, Karnataka, India*

[b] *Sri Siva Subramaniya Nadar College of Engineering, Tamil Nadu, India*

* Corresponding author: Sudha D. Kamath, email: sudha.kamath@manipal.edu

Table S1. The relative error in the radius of the dopant concerning the host ions.

Host	CN	R_1(Å)	Dopant	CN	R_2(Å)	D_R (%)
Ba^{2+}	6	1.35	Dy^{3+}	6	0.91	32.5925
Ba^{2+}	6	1.35	Dy^{3+}	8	1.02	-0.7407
Ba^{2+}	8	1.42	Dy^{3+}	6	0.91	51.9366
Ba^{2+}	8	1.42	Dy^{3+}	8	1.02	28.1690
Ba^{2+}	6	1.35	Sm^{3+}	6	0.91	31.6296
Ba^{2+}	6	1.35	Sm^{3+}	8	1.07	-5.6790
Ba^{2+}	8	1.42	Sm^{3+}	6	0.91	49.8239
Ba^{2+}	8	1.42	Sm^{3+}	8	1.07	24.6478

Table S2. The atomic parameters of $Ba_2ZnSi_2O_7$: Dy^{3+}, Sm^{3+} lattice structure.

Atoms	Wyckoff sites	Occupancy	x	y	z
Ba0	8f	0.94000	0.22663	0.04431	0.97618
Zn1	4f	0.50000	0.00000	0.25762	0.25000
Si2	4e	1.00000	0.12134	0.28480	0.64995
O3	4d	1.00000	0.01565	0.34842	0.48984
O4	4c	1.00000	0.07343	0.14711	0.62818

O5	4b	1.00000	0.19456	0.16059	0.27586
O6	4a	0.50000	0.00000	0.35643	0.75000
Dy7	8f	0.30000	0.22663	0.04431	0.97618
Sm8	8f	0.30000	0.22663	0.04431	0.97618

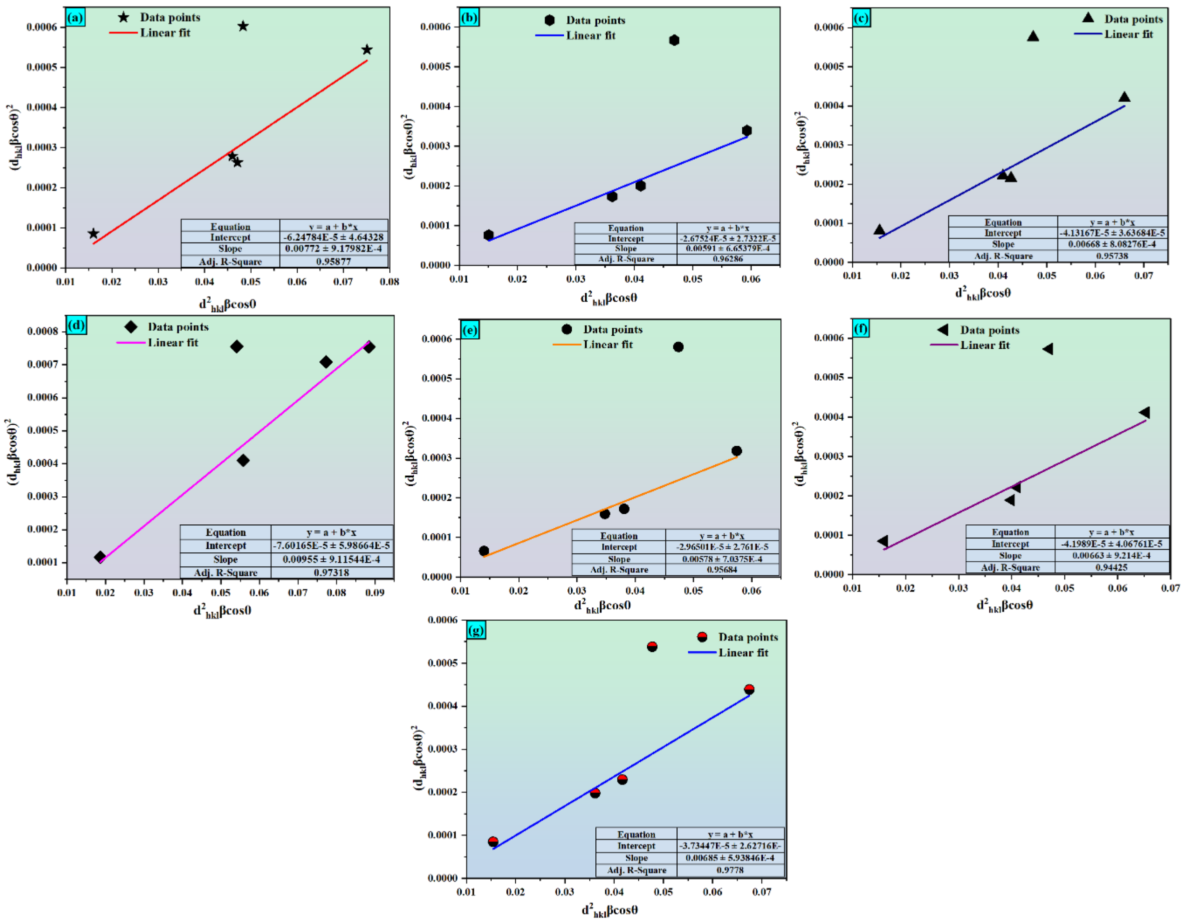

Figure S1 (a-g). Size-Strain plot for the $Ba_{2-x-y}ZnSi_2O_7$: xDy^{3+}, ySm^{3+} phosphors.

Table S3. Crystallite size and strain of the $Ba_{2-x-y}ZnSi_2O_7$: xDy^{3+}, ySm^{3+} phosphors by Debye-Scherrer, and Size-strain plot.

**Dy^{3+} concentrations (mol%)**	**Sm^{3+} concentrations (mol%)**	**Debye-Scherrer method**	**Size-Strain plot**	
		**D_{D-S} (µm)**	**D_{S-S} (µm)**	**ε_{S-S} ($\times 10^{-3}$)**
0	0	0.0199	0.0211	-1.4937

1.5	0	0.0200	0.0187	-24.9914
1.5	0.2	0.0252	0.0244	-10.7010
1.5	0.5	0.0272	0.0151	-30.4066
1.5	1	0.0169	0.0250	-11.8600
1.5	2	0.0261	0.0218	-16.7956
1.5	3	0.0229	0.0216	-16.5267

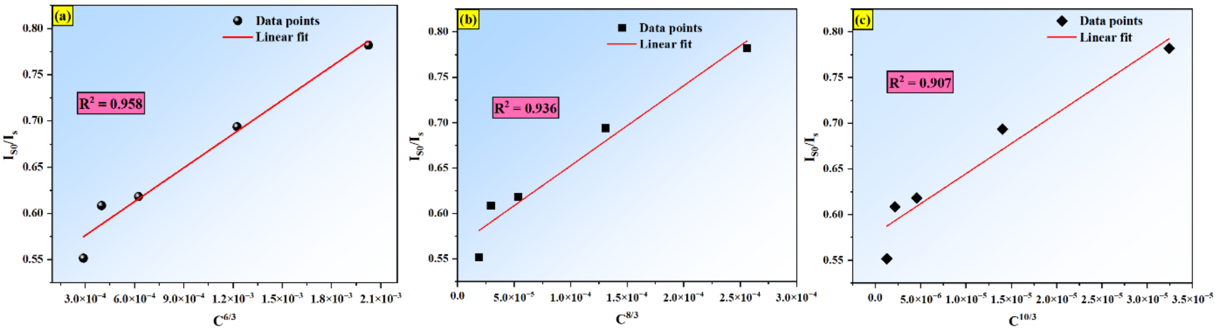

Figure S2 (a-c). Dependence of I_{so}/I_s on (a) $C^{6/3}$, (b) $C^{8/3}$ and (c) $C^{10/3}$.

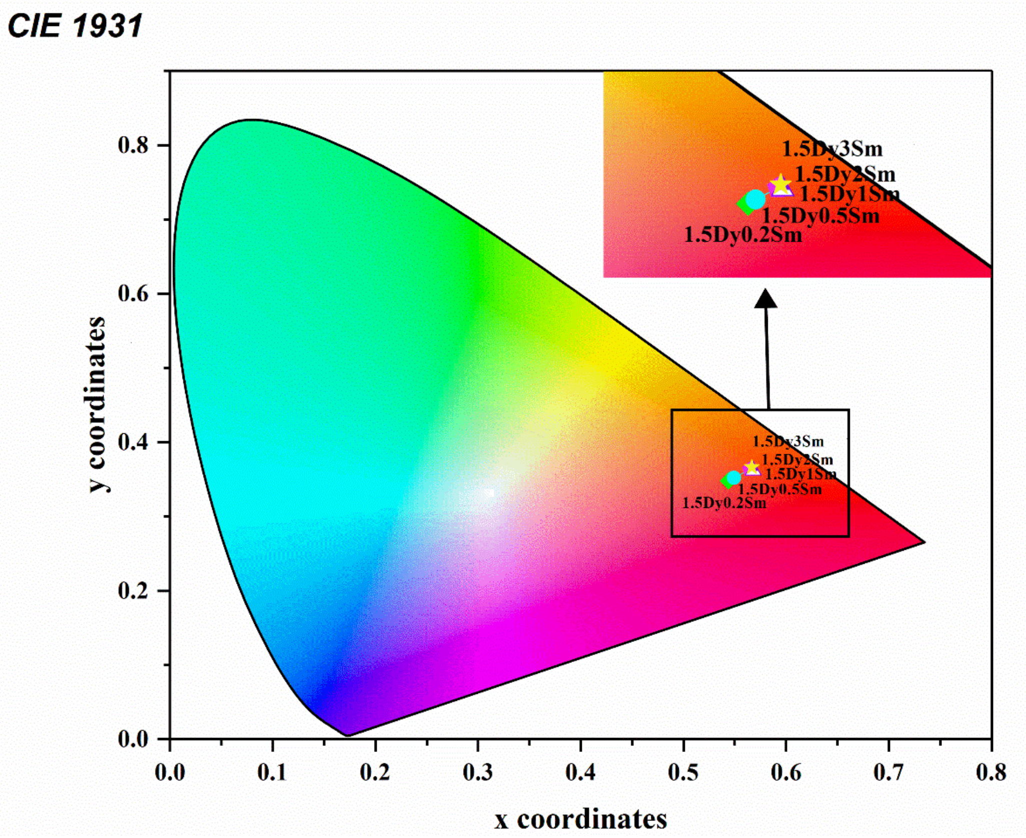

Figure S3. CIE 1931 chromaticity diagram showing the emission coordinates of $Ba_2ZnSi_2O_7$: Dy^{3+}, Sm^{3+} phosphor. The calculated CIE coordinates are (x = 0.56, y = 0.36), with a correlated color temperature (CCT) of 1464 K and a color purity of 78.5%.

Table S4. CIE chromaticity coordinates (x_p, y_p), dominant wavelength (λ_d), color purity (C.P), and CCT values for $Ba_{2-0.15-y}ZnSi_2O_7$: 1.5 mol% Dy^{3+}, y Sm^{3+}(y = 0.2, 0.5, 1.0 2.0, and 3.0 mol%).

**Sm^{3+} concentrations (mol%)**	**(x_p, y_p)**	**(x_d, y_d)**	**λ_d(nm)**	**C.P (%)**	**CCT (K)**
**0.2**	(0.5438,0.3479)	(0.3574, 0.5144)	604.1	67.6	1501
**0.5**	(0.5490,0.3520)	(0.3585, 0.5172)	602.9	70.4	1493
**1.0**	(0.5647,0.3632)	(0.3626, 0.5248)	600.2	78.5	1464
**2.0**	(0.5680,0.3618)	(0.3661, 0.5248)	600.7	79.1	1439
**3.0**	(0.5667,0.3663)	(0.3619, 0.5265)	599.6	80.0	1469

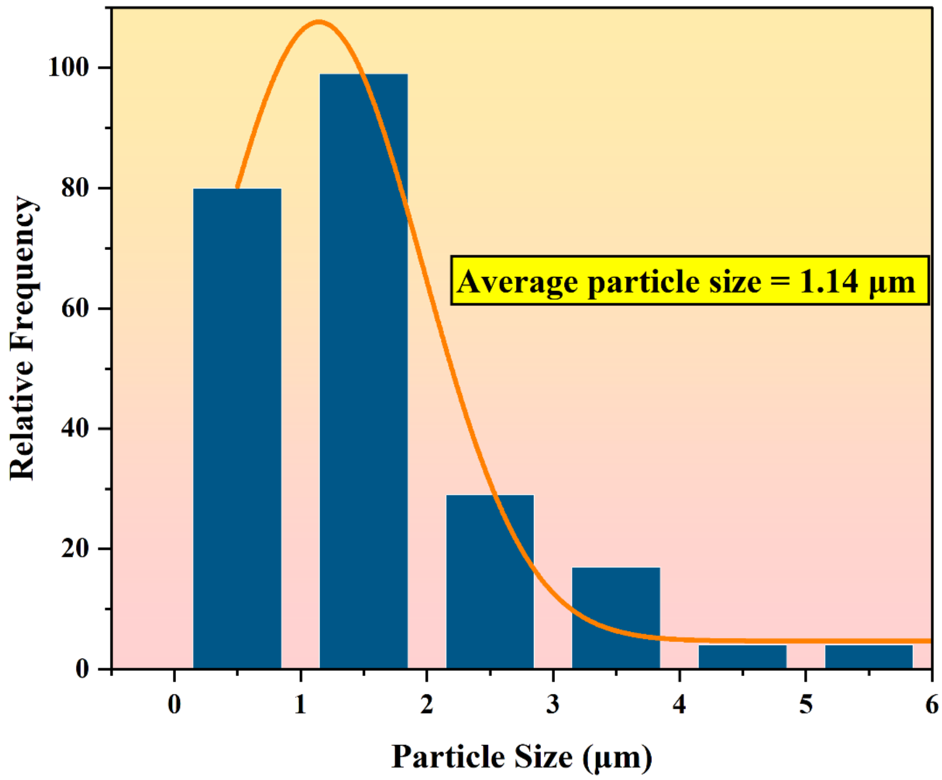

Figure S4. Particle size distribution estimated from SEM images.
